# Eosinophil recruitment is dynamically regulated by interplay among lung dendritic cell subsets after allergen challenge

**DOI:** 10.1038/s41467-018-06316-9

**Published:** 2018-09-24

**Authors:** Shuying Yi, Jing Zhai, Rui Niu, Guangming Zhu, Meixiang Wang, Jianguo Liu, Hua Huang, Yaping Wang, Xiuli Jing, Li Kang, Wengang Song, Yufang Shi, Hua Tang

**Affiliations:** 1 Institute of Immunology, Taishan Medical University, Taian, 271000 Shandong China; 2 School of Basic Medical Sciences, Taishan Medical University, Taian, 271000 Shandong China; 3 Shanghai Public Health Clinical Center, Fudan University, Shanghai, 201508 China; 4 Institute of Health Sciences, Shanghai Institutes for Biological Sciences, Shanghai, 200031 China

## Abstract

Eosinophil infiltration, a hallmark of allergic asthma, is essential for type 2 immune responses. How the initial eosinophil recruitment is regulated by lung dendritic cell (DC) subsets during the memory stage after allergen challenge is unclear. Here, we show that the initial eosinophil infiltration is dependent on lung cDC1s, which require nitric oxide (NO) produced by inducible NO synthase from lung CD24^−^CD11b^+^ DC2s for inducing CCL17 and CCL22 to attract eosinophils. During late phase responses after allergen challenge, lung CD24^+^ cDC2s inhibit eosinophil recruitment through secretion of TGF-β1, which impairs the expression of CCL17 and CCL22. Our data suggest that different lung antigen-presenting cells modulate lung cDC1-mediated eosinophil recruitment dynamically, through secreting distinct soluble factors during the memory stage of chronic asthma after allergen challenge in the mouse.

## Abstract (web summary)

Eosinophils are important mediators of allergic responses, but how they are recruited to the inflamed site is still unclear. Here the authors show that CD103^+^ cDC1 cells secrete CCL17 and CCL22 for eosinophil recruitment, with this process promoted by CD24^−^CD11b^+^ DC2s in the early phase but suppressed by CD24^+^ cDC2s in the late phase.

## Introduction

Allergic inflammatory asthma is a common disease that affects people worldwide^1,2^. It is mediated by several varieties of immune cells. Infiltration of eosinophils into the lung from the bone marrow and blood is the hallmark of eosinophilic allergic asthma^1,3,4^. Eosinophils are primarily considered terminally differentiated effector cells, but emerging data supports that eosinophils play a causal role in the augmentation of broader inflammation^1,4–8^. Targeting therapeutics to eosinophils has proved successful in controlling asthma in clinical trials^1,2,4,9–11^.

Eosinophil regulated by several cells, cytokines, and chemokines. IL-5 is essential for the expansion and mobilization of eosinophils from the bone marrow into the lung following allergen exposure^3,12^. CCL11 (eotaxin-1) and CCL24 (eotaxin-2) are the main chemokines involved in eosinophil recruitment^3,12^. Type 2 innate lymphoid cells (ILC2s) have been suggested to be potent inducers of eosinophil migration, either through their production of IL-5 or potentially through the production of CCL11^1,13–15^. Upon allergen challenge, Th2 lymphocytes may produce large amounts of IL-5^11,16^. However, other influencers of eosinophil accumulation in the lung are not yet fully elucidated.

Numerous studies have highlighted the involvement of dendritic cells (DCs) in the development of eosinophilic airway inflammation and asthma^1,5,17^. CD103^+^cDC1s and CD11b^+^ cDC2s are two major lung CD11c^+^ DC subsets. The division of labor among lung DC subsets is increasingly being recognized, with each subset showing both specific and overlapping functions^18–20^.

cDC1s have been shown to be involved in polarization toward Th1 and inhibition of Th2 responses via constitutive expression of IL-12^21,22^. A role for lung cDC1s in promoting Th2 response to inhaled allergens has also been demonstrated^23,24^, although contrary evidence has emerged from recent studies suggesting that cDC1s are not required for eosinophil infiltration during the primary immune response^25,26^. It remains necessary to determine whether lung cDC1s are or are not essential for eosinophil recruitment after allergen challenge.

cDC2s have been shown to be the dominant DC subset involved in promoting eosinophil infiltration during the primary immune response in acute allergic asthma^25,27–30^. However, whether and how cDC2s were involved in regulating eosinophil infiltration during immunological memory phase in chronic allergic asthma is still unclear. Furthermore, the necessity of professional APCs, including DCs, during the memory stage in chronic eosinophilic asthma^31,32^ has been challenged by a published study in which memory Th2 cells were responsible for IL-33-mediated exacerbations of eosinophilic inflammation in a MHC II-independent manner^33^.

In our current study, we show that in a chronic allergic asthma mouse model focused on the memory stage after allergen challenge, the initial eosinophil recruitment is mediated by cDC1s, which directly attract eosinophils by secreting CCL17 and CCL22. Furthermore, our data support the notion that cDC1-mediated eosinophil infiltration is dynamically modulated by other lung DC subsets. On day 1.5 after the first allergen challenge, lung CD24^−^CD11b^+^ DC2s promote eosinophil infiltration via producing nitric oxide (NO), whereas CD24^+^ cDC2s inhibit this process by releasing TGF-β1 on day 2.5.

## Results

### Lung CD11c^+^ DCs are required for eosinophil recruitment

**Fig. 1:**
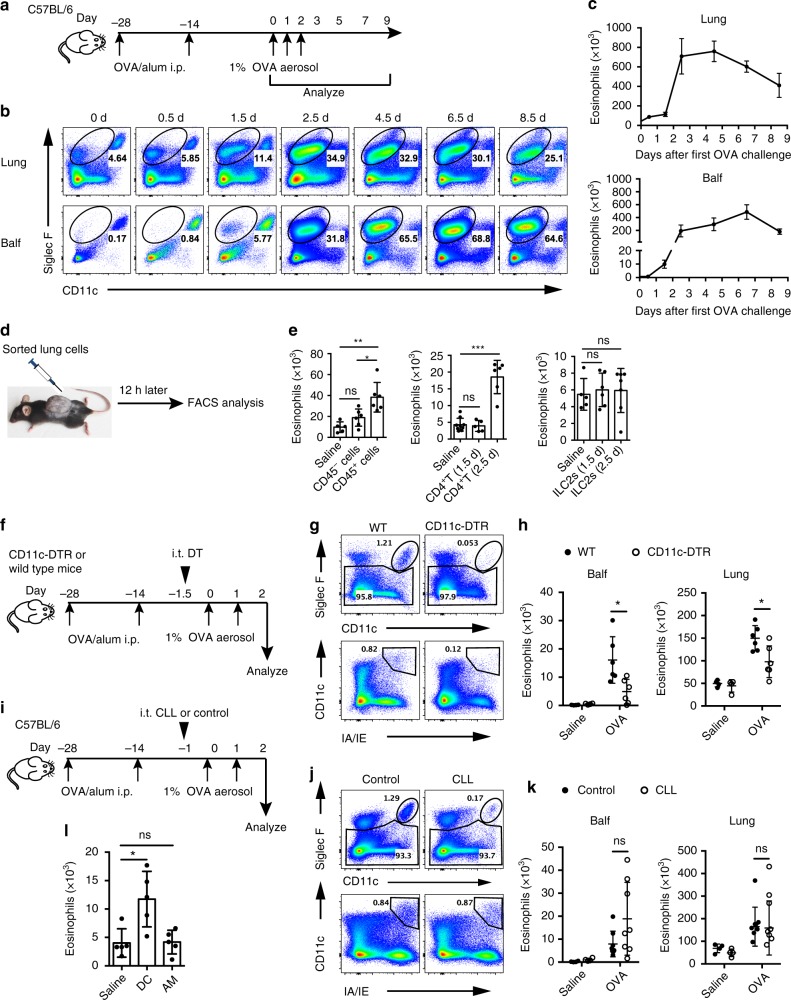
Lung DCs are required for eosinophil recruitment during allergen challenge. **a** Mice model of kinetics of eosinophil recruitment. **b**, **c** FACS (**b**) and total numbers (**c**) of eosinophil recruitment in different lung compartments in the murine kinetics model of allergic inflammation shown in **a**. Upper row, lung tissue homogenates; lower row, Balf. *n* = 4–8 mice per group. **d** Protocol for in vivo recruitment assay (air-pouch assay). **e** Counts of eosinophils recruited into the air pouches of wild-type (WT) mice injected with lung CD45^−^ cells or CD45^+^ cells (left), lung CD4^+^ T cells (middle), or ILC2s (right) were analyzed by FACS. *n* = 5–7 mice per group. **f**–**h** Eosinophil recruitment of allergic CD11c-DTR Tg (empty circle) or WT (solid circle) mice i.t. injected with DT to delete lung CD11c^+^ DCs and AMs, where **f** shows the protocol. **g** Different deletion efficiencies of DCs and AMs in the lung of *CD11c-DTR* Tg or WT mice after i.t. instillation with DT. **h** Total numbers of eosinophils in the lung or Balf. *n* = 4 mice (Saline) or *n* = 6 mice (OVA). **i**–**k** Eosinophil recruitment of allergic C57Bl/6 mice i.t. injected with clodronate liposome (CLL) (empty circle) or control (solid circle) on day −1.5 to delete lung AMs, where **i** presents the protocol. **j** 24 h after CLL or empty liposome instillation, lungs were analyzed for the presence of AMs. **k** The total numbers of eosinophils in the lung or Balf. *n* = 4 mice (Saline) or *n* = 8 mice (OVA). **l** Counts of eosinophils recruited into the air pouches of WT mice injected with pulmonary SiglecF^−^CD11c^+^IA/IE^+^ DCs or SiglecF^+^CD11c^high^ AMs (5 × 10^4^ cells, 200 μl) from OVA-challenged mice. *n* = 4–5 mice per group. *P < 0.05, **P < 0.01, ***P < 0.001, unpaired Student’s *t* test. Means ± SD are shown. Data represent two (**a**–**c**) and three (**d**–**i**) independent experiments

To investigate eosinophil recruitment in the lung during memory stage after allergen challenge in a chronic mice model, we employed a kinetics analysis. Mice were sensitized with ovalbumin (OVA)/aluminum hydroxide (alum) by intraperitoneal (i.p.) injection and 28 days later challenged intranasally (i.n.) with OVA aerosol as the times indicated in Fig. 1a. Eosinophil infiltration in the lung and bronchoalveolar lavage fluid (Balf) were assayed at indicated time points by fluorescence-activated cell sorting (FACS). In the lung and Balf, eosinophils started to accumulate as early as 1.5 days after the first OVA challenge (Fig. 1a–c). This result was consistent with the previous work^34^.

It has been suggested that the initial eosinophil recruitment is required for the induction of a type 2 immune response to OVA aerosol challenge^8,35^. This was also confirmed by our data (Supplementary Fig. 1). However, how this early eosinophil infiltration is initiated has been largely unknown. We hypothesized that during the early eosinophil infiltration phase of OVA challenge, some lung cells may secrete certain chemokines to recruit eosinophils. To determine which cell type in the lung is capable to recruit eosinophils, we did an air-pouch assay as described^36,37^. On day 1.5 after the first inhaled OVA challenge, CD45^+^ or CD45^−^ cells sorted from lungs were injected into mouse air pouches that had been prepared in advance in naive recipients. Twelve hours later, eosinophils in the air pouches were assayed by FACS (Fig. 1d, Supplementary Fig. 2). The results showed that lung CD45^+^ cells, but not CD45^−^ cells, from OVA-challenged mice exhibited activity to recruit eosinophils (Fig. 1e). The results were also confirmed by transwell assay (Supplementary Fig. 3), suggesting that CD45^+^ hematopoietic cells recruit eosinophil directly.

Then we sought to determine among CD45^+^ cells which cell type could recruit eosinophils. Both CD4^+^ T cells and ILC2s have been proposed to play important roles in regulating eosinophil recruitment^11,13,15^. Memory Th2 cells are known to promote eosinophil infiltration through MHC II-independent paths^33^, which was confirmed by our results (Supplementary Fig. 4a, b). Ablation of CD4^+^ T cells resulted in significantly reduced eosinophil infiltration (Supplementary Fig. 4c–e), as reported in a previous study^33^. However, in the air-pouch assay, no eosinophil chemotactic effect was displayed by lung CD4^+^ T cells from mice on day 1.5 after the first OVA challenge (Fig. 1e), although the effect was displayed on day 2.5 after challenge (Fig. 1e). We also failed to observe lung ILC2s display any eosinophil recruitment competency both on day 1.5 and day 2.5 after challenge (Fig. 1e). These data collectively demonstrated that neither CD4^+^ T cells nor ILC2s were capable to recruit eosinophils by themselves on day 1.5 after the first challenge during the memory stage.

Given that the localization of DCs and alveolar macrophages (AMs) were closely associated with the airway and alveoli^38,39^, we hypothesized that DCs or AMs might be required to recruit eosinophils from blood vessels to the inflamed lung and alveoli. To test this, *CD11c-DTR* mice were used, as both DCs and AMs express CD11c. Intratracheal (i.t.) injection of diphtheria toxin (DT) into *CD11c-DTR* mice resulted in efficient depletion of DCs and AMs from the lung (Supplementary Fig. 5 and Fig. 1f, g). Data showed that the number of eosinophils in Balf and lung was significantly reduced in *CD11c-DTR* mice compared with wild-type mice (Fig. 1h). Consistent with previous data^31,32^, our results demonstrated that lung CD11c^+^ APCs are required for the recruitment of eosinophils in response to inhaled OVA challenge.

To further examine which CD11c^+^ APCs subset was involved in eosinophil infiltration, we employed a mouse model in which AMs could be almost completely ablated within 24 h after clodronate liposome (CLL) i.t. injection and leave the lung CD11c^+^ DCs intact (Fig. 1i, j), as has been described before^40^. We found that there was no noticeable change in the eosinophil infiltration in AM-ablated mice on day 1.5 after the first OVA challenge (Fig. 1k). These data demonstrated that OVA challenge-induced eosinophil infiltration was independent of AMs. This suggested that CD11c^+^ DCs were critical for eosinophil recruitment. We also found that the temporal change curve of CD11c^+^ DCs was similar to that of eosinophils both in the lung and Balf (Supplementary Fig. 6). We therefore sought to determine whether CD11c^+^ DCs are capable of inciting eosinophil infiltration in vivo. To test this, lung CD11c^+^ DCs and AMs were sorted on day 1.5 after the first OVA aerosol challenge and injected into the air pouches. Then, 12 h later, we calculated eosinophils in the air pouches with FACS and found that it was CD11c^+^ DCs, but not AMs, that attracted eosinophils in vivo (Fig. 1l). These data strongly suggested that lung CD11c^+^ DCs were essential for the initial eosinophil accumulation during memory stage in chronic asthmatic inflammation after challenge, by recruiting eosinophils directly.

### Lung cDC1s attract eosinophils at early infiltration phase

**Fig. 2:**
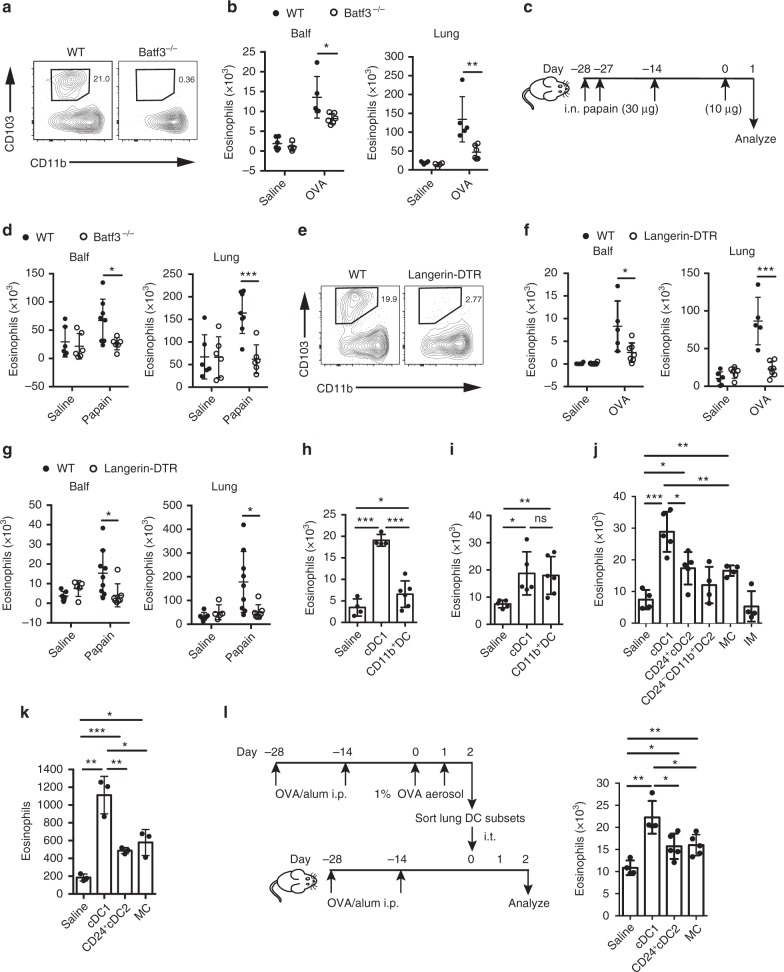
Lung cDC1s are necessary and sufficient for the induction of eosinophilia. **a** Lung cDC1s from WT and *Batf3*^−/−^ mice. **b** Eosinophil counts in the lung or Balf in WT (solid circle) and *Batf3*^−/−^ (empty circle) mice 1.5 days after the first OVA challenge. *n* = 4 or 6 mice (Saline) or *n* = 5–6 mice (OVA). **c**, **d** Mice were sensitized and challenged with papain, and eosinophils in the lung or Balf in WT (solid circle) and *Batf3*^−/−^ (empty circle) mice were analyzed. *n* = 5–7 mice per group. **e** Lung cDC1s from DT treatment *langerin-DTR* or WT mice. **f**, **g** OVA/alum-sensitized (**f**) or papain-sensitized (**g**) *langerin-DTR* (empty circle) or WT (solid circle) mice were treated with DT on day −1, and eosinophils in the lung or Balf were assessed after challenge. *n* = 5–9 mice per group. **h**, **i** Eosinophils recruited into the air pouches injected with pulmonary cDC1s (SiglecF^−^CD11c^+^IA/IE^+^CD103^+^CD11b^−^) or CD11b^+^ DCs (1.5 × 10^4^, 200 μl) sorted from C57BL/6 mice 1.5 days after the first OVA challenge (**h**) or 1 day after the first papain challenge (**i**). *n* = 4–6 mice per group. **j** Eosinophils recruited into the air pouches injected with pulmonary cDC1s, CD24^+^ cDC2s, CD24^−^CD11b^+^ DC2s, MCs, or IMs (1.5 × 10^4^, 200 μl) sorted from C57BL/6 mice 1.5 days after the first OVA challenge. *n* = 4–6 per group. **k** The direct chemotactic effect of lung cDC1s, CD24^+^ cDC2s, or MCs (from C57BL/6 mice 1.5 days after the first OVA challenge) on eosinophils was evaluated in a transwell culture system. *n* = 3–4 per group. **l** Protocol (left) and lung eosinophil counts (right) of allergic airway induction by adoptive i.t. injection of lung cDC1s, CD24^+^ cDC2s, or MCs; see Methods *P < 0.05, **P < 0.01, ***P < 0.001, unpaired Student’s *t* test. Means ± SD are shown. Data represent two (**c**, **d**, **g**, **i**, **l**) and three (**a**, **b**, **e**, **f**, **h**, **j**, **k**) independent experiments

There are two major CD11c^+^ DC subsets in the lung, CD103^+^ DCs (cDC1s) and CD11b^+^ DCs. cDC1s are more closely associated with the airway epithelium than are CD11b^+^ DCs^38^. So we speculated that cDC1s may be more likely to be involved in recruiting eosinophils to the airway if they secrete a certain eosinophil chemotactic factor. To test this hypothesis, we first investigated whether lung cDC1s were required for the initial eosinophil infiltration during memory phase after inhaled allergen challenge. Therefore, we carried out experiments in *Batf3*^−/−^ mice, in which cDC1s were selectively ablated due to severe defects in the development of this population^41^, as indicated in Fig. 2a. We found that after OVA aerosol challenge, the eosinophil infiltration in the Balf and lung from *Batf3*^−/−^ mice was significantly decreased compared with wild type mice, suggesting that cDC1s are required for the initial eosinophil accumulation (Fig. 2b). To confirm that these results were not specific to the OVA model, we performed experiments in a different established mouse model of asthma, in which inhaled cysteine protease papain was used as an allergen (Fig. 2c). As shown in Fig. 2d, similarly, during memory stage after papain challenge, the eosinophil infiltration in the Balf and lung from *Batf3*^−/−^ mice was significantly reduced. To ablate cDC1s immediately before the challenge, *langerin-DTR* mice were used, as cDC1s express langerin. Lung cDC1s could be selectively depleted in *langerin-DTR* mice within 24 h of the injection of DT (Fig. 2e, Supplementary Fig. 7). Mice were challenged 1 day after DT treatment with OVA aerosol. We found that treatment with DT resulted in significantly decreased eosinophil numbers both in the lungs and in Balf (Fig. 2f). Similarly, depletion of lung cDC1s in *langerin-DTR* mice with DT also impaired eosinophil infiltration induced by papain challenge in a papain sensitization/challenge-induced asthma model (Fig. 2g). Collectively, these experiments demonstrated that during the memory stage of chronic asthma, allergen challenge-induced eosinophil infiltration was dependent on lung cDC1s.

To determine whether lung cDC1s from inhaled allergen-challenged mice were capable of recruiting eosinophils in vivo, we sorted lung cDC1s and CD11b^+^ DCs by FACS from mice on day 1.5 after first OVA aerosol challenge, and then injected them into air pouches. Although both lung cDC1s and CD11b^+^ DCs displayed the capacity to recruit eosinophils, cDC1s were much more efficient than CD11b^+^ DCs at recruiting eosinophils (Fig. 2h). To examine whether lung cDC1s from mice challenged with papain were also capable to recruit eosinophils, we performed the similar air pouch experiments. As expected, lung cDC1s from papain challenged mice could recruit eosinophils, although CD11b^+^ DCs displayed similar capacity (Fig. 2i). As lung monocyte-derived cells (MCs) and lung interstitial macrophages (IMs) may also express MHC II and low to intermediate levels of CD11c, it is possible that the lower capacity of CD11b^+^ DCs to attract eosinophils in the air pouch assay was caused by MC or IM contamination. We adopted a recent strategy with a few modifications to identify these different CD11b^+^ cells and exclude MCs and IMs from CD11b^+^ DCs (Supplementary Fig. 8)^42^. Also, recently, it has been suggested that lung CD11b^+^ DCs can be further divided into CD24^+^ cDC2s and CD24^−^ DCs based on CD24 expression^27^. We also tried to divide CD11b^+^ DCs into CD24^+^ and CD24^−^ populations. However, considering that the CD24^−^ population has not been genetically demonstrated as a bona fide flt3l-dependent and maybe IRF4 independent subset^43^, we used CD24^−^CD11b^+^ DC2s to name this population hereafter, although they are phenotypically CD64^−^F4/80^−^Mertk^−^CD11c^+^MHCII^+^CD26^+^Sirpa^+^ and originally CCR2 independent (Supplementary Fig. 9). Considering that the temporal changes of DC subsets (CD24^+^ cDC2s and CD24^−^CD11b^+^ DC2s) in the lung were associated with the kinetic change of eosinophil (Supplementary Fig. 10), we sought to test which CD11b^+^ cell subset (MCs, IMs, CD24^+^ cDC2s, or CD24^−^CD11b^+^ DC2s) is more efficient in recruiting eosinophils in the air pouch assay. Our results showed that lung CD24^+^cDC2s and MCs could recruit eosinophils, but were less efficient than cDC1s (Fig. 2j). We failed to find any chemotactic effects of lung CD24^−^CD11b^+^ DC2s and IMs (Fig. 2j).

To examine whether cDC1s attract eosinophils directly, transwell assays were performed. As shown in Fig. 2k, cDC1s indeed recruited eosinophils directly in vitro and were more efficient than other CD11b^+^ cell subsets. To determine whether lung cDC1s from OVA-challenged mice were sufficient to induce eosinophilia in the lungs, lung cDC1s obtained from OVA aerosol challenged mice were i.t. injected into OVA-sensitized mice, as indicated in Fig. 2l. Control mice received a saline injection. The results showed that adoptive transfer of cDC1s is sufficient to induce eosinophilia in the lungs of sensitized mice compared with those in the saline group (Fig. 2l). In a similar experimental setting, injecting lung CD24^+^ cDC2s and MCs from OVA-challenged mice showed less eosinophil infiltration in the lungs (Fig. 2l). These data suggested that lung cDC1s from OVA-challenged mice during the memory stage were both necessary and sufficient for the induction of lung eosinophilia by directly attracting eosinophils.

### Lung cDC1s recruit eosinophils by CCL17 and CCL22

**Fig. 3:**
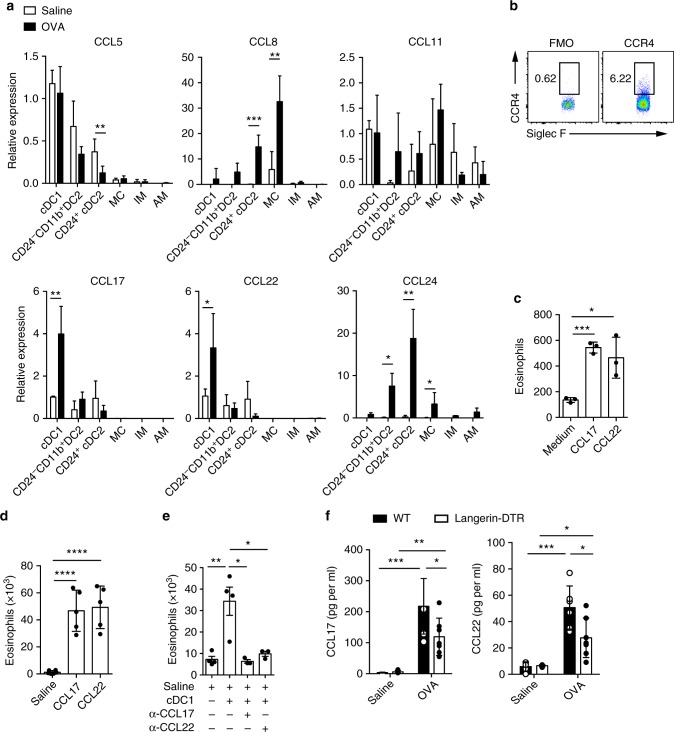
Lung cDC1s directly recruit eosinophils via CCL17 and CCL22. **a** mRNA expression of *CCL5*, *CCL8*, *CCL11*, *CCl17*, *CCL22*, and *CCL24* chemokines by lung cDC1s, CD24^+^ cDC2s, CD24^−^CD11b^+^ DC2s, MCs, IMs, and AMs isolated from mice 1.5 days after the first OVA challenge. mRNA expression was first normalized against the housekeeping GAPDH genes and then represented as relative expression compared to the cDC1s from saline-treated mice. *n* = 3–4 mice per group. **b** FACS analysis of CCR4 expression on lung eosinophils from naive C57Bl/6 mice. **c** The direct chemotactic effect of CCL17 (100 pg per ml) and CCL22 (100 pg per ml) on eosinophils was evaluated in a transwell culture system. *n* = 3 per group. **d** Counts of eosinophils recruited into the air pouches of wild-type mice 5 h after injection of CCL17 (150 pg) or CCL22 (150 pg) with 500 μl of temperature-sensitive surface gel. *n* = 5–6 mice per group. **e** Counts of eosinophils recruited into the air pouches of wild-type mice injected with pulmonary cDC1s (SiglecF^−^CD11c^+^IA/IE^+^CD103^+^CD11b^−^) from OVA-challenged C57Bl/6 mice with CCL17 or CCL22 neutralizing antibody or goat IgG isotype control. *n* = 3–4 mice per group. **f** ELISA analysis of CCL17 and CCL22 in Balf supernatant from DT-treated *langerin-DTR* (empty rectangle) or wild-type (solid rectangle) mice 1.5 days after the first OVA challenge. *n* = 5–7 mice per group. *P < 0.05, **P < 0.01, ***P < 0.001, unpaired Student’s *t* test. Means ± SD are shown. Data represent two (**a**, **b**, **d**, **e**, **f**) and three (**c**) independent experiments

How cDC1s recruit eosinophils remains to be elucidated. The number of cDC1s increased steadily until day 4.5 after OVA challenge (Supplementary Fig. 10a). We hypothesized that lung cDC1s from OVA aerosol challenged mice might secrete chemokines to directly recruit eosinophils. To test this, different subsets of lung DCs and macrophages were sorted from the lungs of OVA- or saline-challenged mice (gating strategy presented in Supplementary Fig. 8) and analyzed for mRNA levels of various chemokines by Q-PCR. cDC1s are not a major source of classical eosinophil recruiting chemokines CCL11 and CCL24. But CCL17 and CCL22 were prominently expressed by lung cDC1s (Fig. 3a), which is in agreement with a published study^25^. It has been demonstrated that CCL17 and CCL22 have the potential to recruit Th2 cells, cutaneous lymphocyte antigen (CLA)-positive skin-homing T cells and Treg cells that express CCR4, a highly specific receptor for those 2 chemokines^44,45^. As CCL17 and CCL22 have been rarely suggested to attract eosinophils^3,46,47^, we sought to test whether these 2 chemokines have the chemotactic effect to recruit eosinophils directly. First, we examined whether CCR4 is expressed on the surface of eosinophils by FACS. We found that CCR4 was indeed expressed on eosinophils (Fig. 3b). Then, both in an air pouch and a transwell system, we observed that CCL17 and CCL22 recruit eosinophils directly (Fig. 3c, d).

To examine whether lung cDC1s from OVA-challenged mice recruit eosinophils via releasing CCL17 and CCL22, air-pouch assay was performed. As shown in Fig. 3e, eosinophil recruitment mediated by cDC1s was completely blocked by anti-CCL17 or anti-CCL22 antibody, suggesting that cDC1s recruit eosinophils by secreting CCL17 and CCL22. However, we observed that neutralization of either CCL17 or CCL22 was sufficient to fully abrogate eosinophil recruitment in vivo. Considering that the concentration of CCL17 or CCL22 produced by injected cDC1s in the air pouches may be very low, this result suggested that physiological CCL17 and CCL22 should synergize to recruit eosinophils. This hypothesis is supported by an experiment (Supplementary Fig. 11) in which low doses of CCL17 and CCL22 were used and injected into air pouches alone or combined. Only the combination group recruited eosinophils, and neither of them attracted eosinophils alone (Supplementary Fig. 11).

We wished to know whether depletion of lung cDC1s could reduce CCL17 and CCL22 levels in the Balf of challenged mice. As expected, on day 1.5 after the first challenge, the level of CCL17 and CCL22 in the Balf was significantly decreased in *langerin-DTR* mice depleted of cDC1s (Fig. 3f). These data suggested that cDC1s may be an important source of CCL17 and CCL22 in the lumen of airways of OVA-challenged mice. Taken together, the above data demonstrated that lung cDC1s recruit eosinophils by secreting CCL17 and CCL22.

### CD24^−^CD11b^+^ DC2s promote eosinophil recruitment by cDC1

**Fig. 4:**
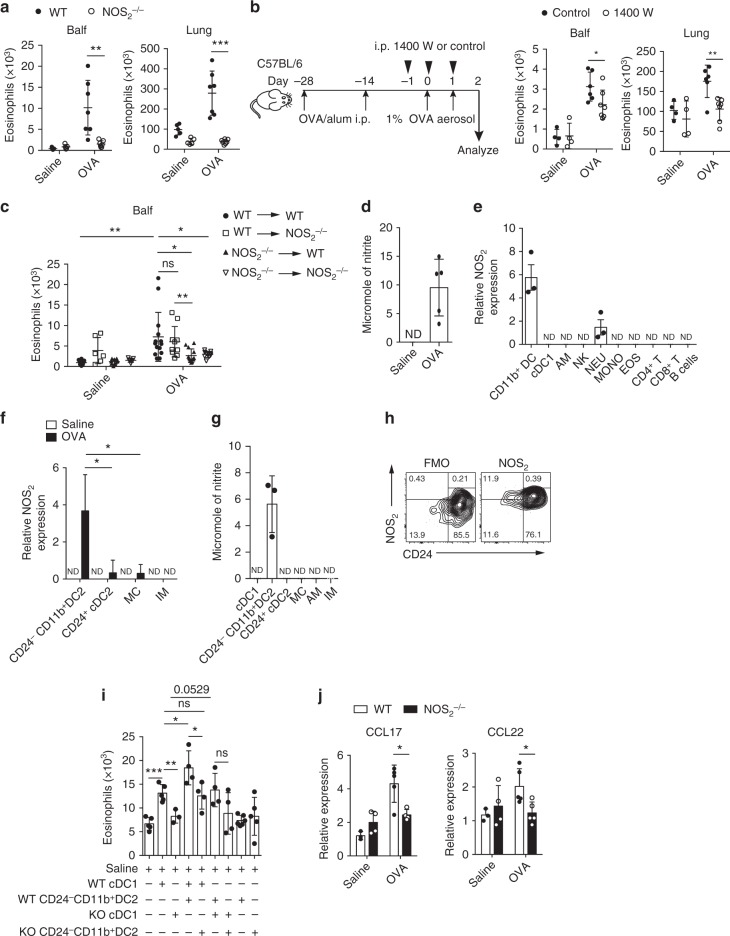
Lung CD24^−^CD11b^+^ DC2s promote cDC1-mediated eosinophil recruitment. **a**–**c** Mice were sensitized and challenged with OVA and culled 1.5 days after the first OVA challenge, and eosinophil counts in the lung or Balf were assessed. **a** WT (solid circle) and *NOS*_2_^−/−^ (empty circle) mice. *n* = 5–7. **b** 1400 W (empty circle) or control-treated (solid circle) mice, protocol on the left. *n* = 4–7. **c** *NOS*_2_^−/−^ and WT bone marrow chimeric mice. WT to WT (solid circle), WT to *NOS*_2_^−/−^ (empty square), *NOS*_2_^−/−^ to WT (solid triangle), and *NOS*_2_^−/−^ to *NOS*_2_^−/−^ (empty triangle) mice. *n* = 6–10 (Saline) or *n* = 10–14 (OVA). **d**–**f** Cells were sorted from mice 1.5 days after the first OVA challenge and used for assay. **d** NO production measured as nitrite in lung CD45^+^ cell culture supernatants. *n* = 4–5 per group. **e** mRNA expression of *NOS*_2_ by different types of lung CD45^+^ cells, such as CD11b^+^DCs, cDC1s (SiglecF^−^CD11c^+^IA/IE^+^CD103^+^CD11b^−^), neutrophils (NEU), etc. *n* = 3 per group. **f** *NOS*_*2*_ mRNA expression in lung CD24^−^CD11b^+^ DC2s isolated from OVA (solid rectangle) or saline (empty rectangle) challenged mice. mRNA expression is shown relative to the expression of NEU (**e**) or CD24^+^ cDC2s (**f**). *n* = 4 per group. **g** NO production measured in lung CD24^−^CD11b^+^ DC2 culture supernatants. *n* = 4 per group. **h** FACS analysis of *NOS*_2_ expression on lung CD11b^+^ DCs from OVA-challenge mice. **i**, **j** Cells were purified from *NOS*_2_^*−/−*^ or WT mice 1.5 days after the first OVA challenge. **i** Eosinophils recruited into the air pouches injected with pulmonary cDC1s (SiglecF^−^CD11c^+^IA/IE^+^CD103^+^CD11b^−^) (1 × 10^4^ cells), CD24^−^CD11b^+^ DC2s (1 × 10^4^ cells). *n* = 4–6 per group. **j** mRNA expression of *CCL17* and *CCL22* by lung cDC1s from *NOS*_2_^*−/−*^ (solid rectangle) or WT mice (empty rectangle). *n* = 3–5 per group. *P < 0.05, **P < 0.01, ***P < 0.001, unpaired Student’s *t* test. Means ± SD are shown. Data represent two (**c**, **e**) and three (**a**, **b**, **d**, **f**–**j**) independent experiments

We sought to investigate how cDC1-mediated eosinophil migration is modulated. It has been suggested that NO plays a key role in allergic asthma. Specifically, NO synthesized by inducible nitric oxide synthase (iNOS, also named NOS_2_) appears to promote eosinophil infiltration into the lungs in an OVA/alum model^48–50^. However, the mechanism by which iNOS-synthesized NO promotes allergic eosinophil influx is poorly understood. To investigate this, we first used *NOS*_2_^−/−^ mice to examine whether inducible NO was regulating eosinophil recruitment in our system. On day 1.5 post first OVA challenge, eosinophil number in both the lungs and Balf of *NOS*_2_^−/−^ mice was significantly decreased (Fig. 4a). To test whether blocking NO production before challenge could inhibit eosinophil infiltration, *N*-[3-(aminomethyl)benzyl] acetamidine (1400 W), a selective iNOS inhibitor, was used. We found that 1400 W treatment significantly suppressed eosinophil infiltration in the Balf and lung (Fig. 4b).

To determine the relative contribution of iNOS-synthesized NO on lung stromal cells versus hematopoietic cells in response to OVA aerosol challenge, we generated radiation-induced chimeric mice, as indicated in Fig. 4c. Eosinophils in the Balf of mice 1.5 days after the first OVA challenge were detected by FACS. We found that expression of iNOS in hematopoietic cells was critical for eosinophil recruitment, since NOS_2_^−/−^→WT chimeric mice failed to recruit eosinophils in response to OVA aerosol challenge (Fig. 4c).

To determine the major source of NO in the lung hematopoietic cells, we sorted lung CD45^+^ cells from OVA-challenged mice and cultured them at 37 °C for 48 h. We found that nitrite was detectable in the supernatant of cultures of CD45^+^ lung cells (Fig. 4d). To determine which hematopoietic cell in the lung was the main NO producer, we purified different types of CD45^+^ cells from the lungs of these mice by FACS and performed Q-PCR assays. As shown in Fig. 4e, we found that CD11b^+^ DCs (SiglecF^−^CD11c^+^IA/IE^+^CD103^−^CD11b^+^) were the major NO producer (Fig. 4e). To determine which lung CD11b^+^ APC subsets produced NO, we purified different CD11b^+^ APC subsets from OVA-challenged mice by FACS and conducted a Q-PCR assay. We found that CD24^−^CD11b^+^ DC2s were the major NO producer (Fig. 4f). By Griess assay, we also found that NO was preferentially produced by CD24^−^CD11b^+^ DC2s, but not by cDC1s, CD24^+^ cDC2s, AMs, or IMs (Fig. 4g). This result was further supported by FACS analysis (Fig. 4h).

Considering that NO promotes eosinophil infiltration and that CD24^−^CD11b^+^ DC2s were the main source of NO in the lung after challenge, we hypothesized that CD24^−^CD11b^+^ DC2s could promote cDC1-mediated eosinophil infiltration via NO. To test this, air-pouch assay was performed as indicated in Fig. 4i. Both WT and KO CD24^−^CD11b^+^ DC2s did not display any capacity to recruit eosinophils. However, compared to WT cDC1s alone, the eosinophil number in the air pouches was significantly increased if WT cDC1s were injected together with WT CD24^−^CD11b^+^ DC2s (Fig. 4i). These data suggested that CD24^−^CD11b^+^ DC2s indeed promoted cDC1-mediated eosinophil infiltration. Interestingly, the results also showed that the WT cDC1-mediated eosinophil recruitment effects can be enhanced by WT CD24^−^CD11b^+^ DC2s but not KO CD24^−^CD11b^+^ DC2s, suggesting that NO from CD24^−^CD11b^+^ DC2s is critical for cDC1-initiated eosinophil infiltration (Fig. 4i). This result further suggested that CD24^−^CD11b^+^ DC2-derived NO is essential for cDC1s to initiate eosinophil recruitment, which is partially supported by the fact that CD24^−^CD11b^+^ DC2s were also significantly depleted in *CD11c-DTR* mice (Supplementary Fig. 12), which showed that lung CD11c^+^ APCs were required for the recruitment of eosinophils in response to OVA challenge. Taken together, the above data demonstrated that CD24^−^CD11b^+^ DC2s indeed promoted cDC1-mediated eosinophil infiltration by producing NO.

We next sought to determine whether the effect of NO might have been achieved through modulating CCL17 and CCL22 expression by lung cDC1s. To test this, the expression of CCL17 and CCL22 in lung cDC1s from NOS_2_^−/−^ mice or wild type mice 1.5 days after the first OVA challenge was analyzed by Q-PCR. As shown in Fig. 4j, CCL17 and CCL22 expression was significantly decreased in lung cDC1s of NOS_2_^−/−^ mice compared to those from control mice. Taken together, these data suggested that lung CD24^−^CD11b^+^ DC2s promote cDC1-mediated eosinophil infiltration through NO.

### Lung CD24^+^ cDC2s inhibit cDC1-mediated eosinophil migration

**Fig. 5:**
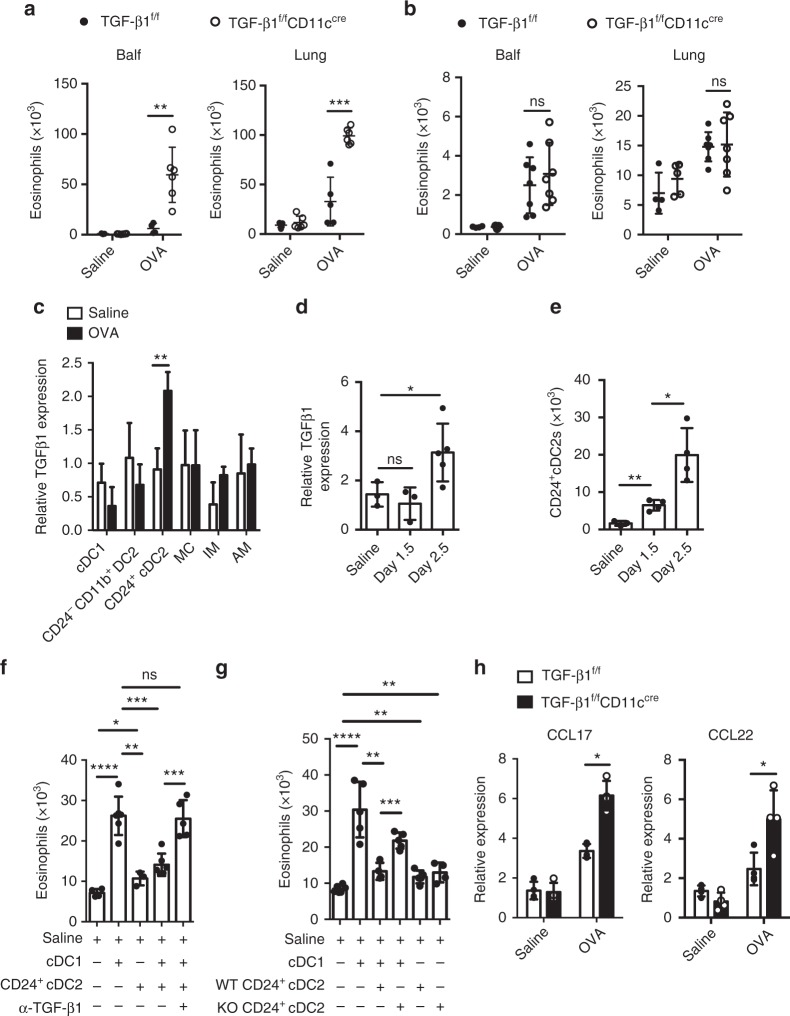
Lung CD24^+^ cDC2s inhibit cDC1-mediated eosinophil migration via TGF-β1. **a**, **b** Quantification of eosinophils in Balf and lung from *TGF-β1*^fl/fl^*CD11c*^Cre^ (empty circle) and *TGF*-*β1*^fl/fl^ (solid circle) mice 2.5 days (**a**, *n* *=* 5–6 mice per group) or 1.5 days (**b**, *n* = 4–5 mice for saline groups and *n* = 7 mice for OVA groups) after the first OVA challenge. **c** mRNA expression of *TGF*-*β1* by pulmonary cDC1, CD24^+^ cDC2, CD24^−^CD11b^+^ DC2, MC, IM and AM populations separated from C57BL/6 mice 2.5 days after the first OVA (solid rectangle) or saline (empty rectangle) challenge. **d** mRNA expression of *TGF-β1* by pulmonary CD24^+^ cDC2s separated from C57BL/6 mice 0, 1.5, or 2.5 days after the first OVA challenge. mRNA expression is relative to the expression of cDC1s (**c**) or CD24^−^CD11b^+^ DC2s (**d**) from saline-treated mice. *n* = 3–5 per group. **e** Counts of CD24^+^ cDC2s in lungs from C57BL/6 mice 0, 1.5, or 2.5 days after the first OVA challenge. **f** Counts of eosinophils recruited into the air pouches injected with pulmonary cDC1s or CD24^+^ cDC2s (1 × 10^4^ cells, 200 μl) purified from C57BL/6 mice 2.5 days after the first OVA challenge with anti-TGF-β1 (10 μg per ml) antibody or mouse IgG1 isotype control. *n* = 4–6 mice per group. **g** Counts of eosinophils recruited into the air pouches injected with pulmonary cDC1s, and CD24^+^ cDC2s (1 × 10^4^ cells, 200 μl) purified from *TGF-β1*^fl/fl^*CD11c*^Cre^ (KO) and *TGF-β1*^fl/fl^ (WT) mice 2.5 days after the first OVA challenge. *n* = 4–6 mice per group. **h** mRNA expression of *CCL17* and *CCL22* chemokines by lung cDC1s (SiglecF^−^CD11c^+^IA/IE^+^CD103^+^CD11b^−^), separated from *TGF*-*β1*^fl/fl^*CD11c*^Cre^ (solid rectangle) and *TGF-β1*^fl/fl^ (empty rectangle) mice 2.5 days after the first OVA challenge. *n* = 4–5 per group. *P < 0.05, **P < 0.01, ***P < 0.001, unpaired Student’s *t* test. Means ± SD are shown. Data represent two (**c**, **d**) and three (**a**, **b**, **e**–**h**) independent experiments

The data shown in Fig. 1c revealed that on day 2.5 after the first OVA challenge there was a turning point, which suggested that an anti-inflammatory factor starts to shut down the inflammation. Considering that *TGF-β1* is an important anti-inflammatory cytokine and that lung CD11c^+^ DCs are critical for eosinophil recruitment, we postulated that if *TGF-β1* was selectively depleted in CD11c^+^ cells, it might lead to increased infiltration of eosinophils on day 2.5 after the first OVA challenge. To test this, *TGF-β1*^fl/fl^*CD11c*^cre^ mice were used in which the increased *TGF-β1* expression can be selectively downregulated in lung CD11c^hi^ cells (Supplementary Fig. 13). On day 2.5 after the first OVA aerosol challenge, mice with CD11c^+^ cell-specific loss of *TGF-β1* showed augmented eosinophil infiltration (in lung and Balf) compared with the *TGF-β1*^fl/fl^ controls (Fig. 5a). Importantly, this effect was not seen on day 1.5 (Fig. 5b).

Next, we sought to determine which CD11c^+^ cells in lung could upregulate the *TGF-β1* expression. As shown in Fig. 5c, the data revealed that the expression of *TGF-β1* in CD24^+^ cDC2s was significantly upregulated, but not in other DC or macrophage subsets (Fig. 5c). And the induced expression of *TGF-β1* in CD24^+^ cDC2s was only observed on day 2.5, but not on day 1.5, although the cell number of CD24^+^ cDC2 was increased steadily after OVA aerosol challenge (Fig. 5d, e). Then, we sought to determine whether CD24^+^ cDC2s inhibit cDC1-mediated eosinophil infiltration, and if so, whether this inhibitory effect is mediated by TGF-β1. For this investigation, mice were culled on day 2.5 after the first OVA challenge, and lung CD24^+^ cDC2s with cDC1s were put together in air pouches. We found the cDC1-mediated eosinophil infiltration in the air pouches was significantly reduced (Fig. 5f). Notably, this inhibitory effect could be reversed by adding α-TGF-β1 antibody into an air pouch (Fig. 5f). These data demonstrated that the inhibitory effect of CD24^+^ cDC2s on cDC1-mediated eosinophil recruitment was mediated by TGF-β1. To further confirm this, we injected CD24^+^ cDC2s from *TGF-β1*^fl/fl^*CD11c*^cre^ mice or *TGF-β1*^fl/fl^ mice together with cDC1s into the air pouches. We found that the CD24^+^ cDC2 loss of *TGF-β1* did not suppress the cDC1-mediated eosinophil infiltration anymore (Fig. 5g). We also found that *CCL17* and *CCL22* production by lung cDC1s from *TGF-β1*^fl/fl^*CD11c*^cre^ mice significantly increased compared to control mice on day 2.5 after the first OVA aerosol challenge (Fig. 5h), indicating that the expression of CCL17 and CCL22 was regulated by TGF-β1 from CD24^+^ cDC2s. Taken together, our data suggested that during the late eosinophil infiltration phase after inhaled OVA challenge (day 2.5 after the first OVA aerosol challenge), lung CD24^+^ cDC2s suppressed lung cDC1-mediated eosinophil infiltration by secretion of TGF-β1.

**Fig. 6:**
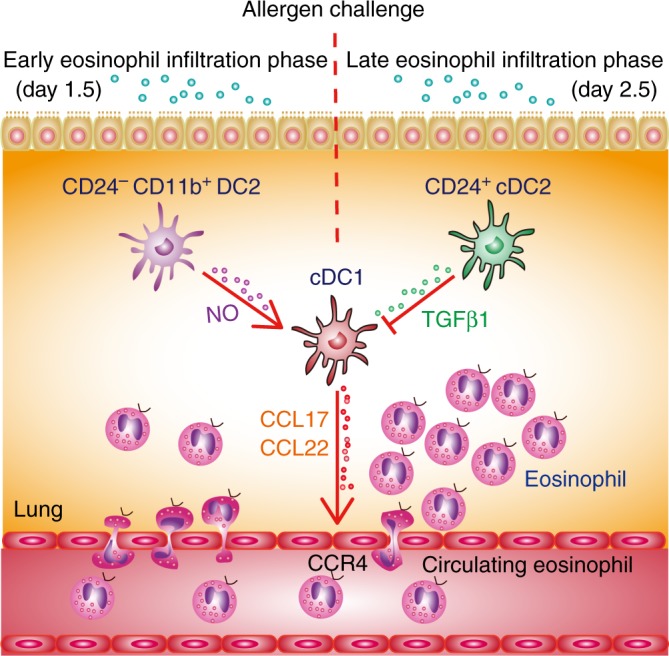
Initial eosinophil recruitment is dynamically regulated by lung DC subsets. After allergen challenge, cDC1s directly recruit eosinophils by secreting CCL17 and CCL22, which are critical for early eosinophil infiltration. This cDC1-mediated eosinophil infiltration is dynamically modulated by other lung DC subsets. After allergen challenge, lung CD24^−^CD11b^+^ DC2s promoted eosinophil infiltration by producing NO on day 1.5, which promotes *CCL17* and *CCL22* expression by cDC1s, whereas CD24^+^ cDC2s inhibit this process by releasing TGF-β1 on day 2.5

Collectively, these data reveal that different lung DC subsets modulate cDC1-mediated eosinophil recruitment dynamically by secreting distinct soluble factors during the memory stage of chronic asthma after allergen challenge in mice (Fig. 6).

## Discussion

How eosinophils are initially recruited to the lung tissue and airway during the memory phase of chronic allergic asthma after allergen challenge is largely unknown. Although both CD4^+^ T and ILC2s appear to play roles in eosinophilic asthma^5,51^, neither of them shows any capacity to attract eosinophil by themselves on day 1.5 after the first OVA challenge (Fig. 1e). Our results showed that DCs are capable of directly attracting eosinophils, and they are essential for the initial eosinophil accumulation after allergen challenge.

Our results demonstrated that in a chronic asthma model, eosinophil infiltration was impaired in cDC1-deficient *Batf3*^−/−^ mice in response to inhaled OVA or papain challenge. Depletion of cDC1s immediately before the challenge in *langerin-DTR* mice also decreased eosinophil infiltration both in OVA/alum or papain-induced chronic asthma during the memory stage. The fact that cDC1s were essential for type 2 airway inflammation in response to OVA or papain allergen challenge seems to contradict a prior publication^25,26^, which showed that during the challenge phase cDC1s are not required for dust mite (*Blomia tropicalis* or *Dermatophagoides pteronyssinus*)-induced allergic airway inflammation. The explanation for these discrepancies could be that the experimental models are fundamentally different, with different sensitization/challenge protocols performed. In the acute “dust mite model”^25,26^, cDC1s were ablated by DT treatment in naive *langerin-DTR* animals before allergen sensitization, and then animals were challenged on day 7 or 9 after the first sensitization, a time point at which more than 64% of cDC1s were recovered from depletion (Supplementary Fig. 7). Thus, these results actually reflect the contribution of cDC1s to the primary immune response. On the other hand, in the current study, a chronic model was used, and mice were challenged 28 days later after the first sensitization, and the influence of cDC1s on eosinophil infiltration on day 1.5 after the first challenge was examined, which reflects the role of cDC1s in memory phase. Thus, it is likely that CD11b^+^ DCs are essential for the primary Th2 response, while cDC1s are critical for eosinophil infiltration during memory stage after challenge.

More interestingly, our data showed that lung cDC1s from challenged mice can directly recruit eosinophils. Further, we found that cDC1 subsets from OVA aerosol challenged mice were sufficient to induce eosinophilia in the lungs of OVA-sensitized mice, which was consistent with published results in which adoptive transfer of DCs is sufficient to induce all asthmatic features in sensitized mice^32^.

We found that lung cDC1s expressed high levels of *CCL17* and *CCL22*, but low levels of *CCL11* and *CCL24* expression, which is consistent with the published literature^25^. Classically, eosinophil recruitment is thought to be driven by eotactic chemokines such as CCL11 and CCL24^1,3,12^. CCL17 and CCL22 are rarely reported involved in the recruitment of eosinophil. To explore this further, we undertook a series of experiments. First, we demonstrated that CCR4 is expressed on eosinophils, and CCL17 and CCL22 could recruit eosinophils directly (Fig. 3b–d). Then, we found that blocking CCL17 and CCL22 decreased the eosinophil-recruitment activity of lung cDC1s from allergen-challenged mice (Fig. 3e). Third, we demonstrated that after OVA aerosol challenge, the level of CCL17 and CCL22 in the Balf was significantly decreased in mice depleted of cDC1s (Fig. 3f). We found that lung CD24^+^ cDC2s and MCs, with the expression of *CCL24* (Fig. 3a), displayed the capacity to recruit eosinophils, although their capacity was less than that of cDC1s (Fig. 2j, k). However, while *CCL24* was also expressed by CD24^−^CD11b^+^ DC2s (Fig. 3a), they did not show any chemotactic effects (Fig. 2j). A similar phenomenon has also been described in a previous study, in which AM-derived CCL24 is dispensable for IL-13-induced airway inflammation^52^. Although we do not fully understand the reason, it is worth further investigation.

Nevertheless, our data demonstrated a previously unappreciated mechanism that in response to allergen challenge, lung cDC1s attract eosinophils directly through secreting CCL17 and CCL22 during the memory stage in chronic asthma. Considering that eosinophils have been shown to be required for Th2 cell infiltration after allergen challenge via stimulating the production of CCL17 and CCL22 by the lungs^8,35^, we propose a model in which, in response to allergen challenge, cDC1s provide the initial CCL17 and CCL22 to recruit the eosinophil infiltration within 2 days, which in turn stimulated more CCL17 and CCL22 production by the lungs to attract Th2 effector cells.

In the current study, we also found that CD24^−^CD11b^+^ DC2s are capable of producing NO, which plays a key role in promoting eosinophil recruitment after challenge in allergic asthma^48–50^. By using radiation-induced chimeric mice, we found NO from hematopoietic cells, but not from lung stromal cells, was critical for eosinophil recruitment (Fig. 4c). More importantly, we found that the CD24^−^CD11b^+^ DC2s population is the major source of NO in the lungs at the early time point after the first challenge (Fig. 4f–h). By air-pouch assay, we demonstrated that lung CD24^−^CD11b^+^ DC2s from OVA-challenged mice were able to promote cDC1-mediated eosinophil infiltration in vivo via NO (Fig. 4i). Moreover, we proved that NO can act via promoting *CCL17* and *CCL22* expression in cDC1s (Fig. 4j). Therefore, our data not only revealed a previously unrecognized mechanism of the regulation of lung CD24^−^CD11b^+^ DC2s in promoting cDC1-mediated eosinophil recruitment, but also may provide a long-sought explanation for the correlation of exhaled NO and eosinophilic airway inflammation in asthmatic humans^53,54^, and for how NO exacerbates eosinophilic airway inflammation.

Interestingly, we also found CD24^+^ cDC2s, another subset of CD11b^+^ DCs, could negatively regulate eosinophil recruitment during the late eosinophil infiltration phase after challenge by expressing TGF-β1 (Fig. 5). We found that it was CD24^+^ cDC2s, but not other DC subsets or AM, that significantly upregulated *TGF-β1* expression on day 2.5 after the first OVA challenge (Fig. 5c). Mice specifically lacking CD11c^+^ DC-derived TGF-β1 displayed no influence on eosinophil recruitment at the early eosinophil infiltration phase after challenge, but had severely augmented eosinophil recruitment at late eosinophil infiltration phase (Fig. 5a, b). By air-pouch assay, we found that CD24^+^ cDC2s inhibited the cDC1-mediated eosinophil infiltration. Further, we demonstrated that this suppressive effect could be rescued by anti-TGF-β1 antibody treatment (Fig. 5f). To further confirm this, CD24^+^ cDC2s from *TGF-β1*^fl/fl^*CD11c*^cre^ mice were injected together with cDC1s into the air pouches. The data showed that the CD24^+^ cDC2 loss of *TGF-β1* could not suppress the eosinophil infiltration anymore (Fig. 5g). These data demonstrated that the inhibitory effect of CD24^+^ cDC2s on cDC1-mediated eosinophil recruitment was mediated by TGF-β1. Thus, our data revealed a previously unrecognized function for lung CD24^+^ cDC2s as negative regulators to inhibit lung cDC1-mediated eosinophil recruitment by TGF-β1 during the late eosinophil infiltration phase after challenge.

The results of our current study provide several novel insights into how lung DC subsets contribute to the eosinophil initial migration during memory phase after challenge in a chronic allergic asthma animal model. We found that cDC1s directly recruit eosinophils by secreting CCL17 and CCL22, which are critical for early eosinophil infiltration. Furthermore, cDC1-mediated eosinophil infiltration is dynamically modulated by other lung DC subsets. After allergen challenge, lung CD24^−^CD11b^+^ DC2s promote eosinophil infiltration by producing NO on day 1.5, which promoted *CCL17* and *CCL22* expression by cDC1s, whereas CD24^+^ cDC2s inhibit this process by releasing TGF-β1 on day 2.5 (Fig. 6). These insights may facilitate the development of eosinophil-targeted therapeutic approaches for human asthma^4^.

## Methods

### Mice

*CD11c-DTR*, *Batf3*^−/−^, *langerin-DTR*, *NOS*_2_^−/−^, *CD11c*^Cre^, and *TGF-β1*^fl/fl^ mice were purchased from Jackson Laboratories. We generated mice lacking *TGF-β1* in CD11c^+^ cells^55,56^. In brief, CD11c^Cre^ mice were crossed with *TGF-β1*^fl/fl^ mice to generate *TGF-β1*^fl/fl^*CD11c*^Cre^ mice in which *TGF-β1* gene exon6 was specifically depleted in CD11c^+^ cells. The littermate *TGF-β1*^fl/fl^ mice were used as a control. Female C57BL/6 (B6; H-2 Kb) mice, 6–8 weeks of age, were purchased from Vitalriver (Beijing, China). Mice were maintained in specific pathogen-free condition, and all studies were approved by the Laboratory Animal Care Committee of Taishan Medical University.

### Reagents

Ovalbumin (OVA) grade V and grade VI, papain, and diphtheria toxin (DT) were from Sigma Aldrich. Imject Alum (aluminum hydroxide) was from Pierce, Rockford, IL. Carboxyfluorescein succinimidyl ester (CFSE) was purchased from Invitrogen. The RNA Mini kit for RNA isolation and RT-PCR kit were from Qiagen. Collagenase type IV and ACK buffer were purchased from Gibco Life Technologies. EDTA was from Invitrogen. N-[3 (aminomethyl) benzyl] acetamidine (1400 W) was from Santa Cruz. Percoll and fetal bovine serum (FBS) were purchased from GE Healthcare. RPMI-1640 medium was from Thermo Fisher Scientific. Clodronate liposomes (CLLs) were prepared as previously described^57^. Briefly, 1% of the dichloromethylene bisphosphonate (Cl_2_MBP) could be encapsulated in the liposomes, and the final (Cl_2_MBP)–liposome suspension (4 ml) contained approximately 20 mg of (Cl_2_MBP).

### Antibodies

The following reagents were purchased from BD: anti-Siglec F (Clone: E50-2440, Cat: 552126 and 562681, dilution 1:200) and biotin-labeled anti-CD103 (Clone: M290, Cat: 557493, dilution 1:400). The following were purchased from BioLegend: anti-CD64 (Clone: X54-5/7.1, Cat: 139306, dilution 1:200), anti-CD24 (Clone: M1/69, Cat: 101816, dilution 1:200), anti-MHCII (Clone: M5/114.15.2, Cat: 107630 and 107635, dilution 1:800 or 1:200), anti-ly6G (Clone: 1A8, Cat: 127624, dilution 1:800), anti-CCR4 (Clone: 2G12, Cat: 131217, dilution 1:200), and Brilliant Violet 650^TM^ Streptavidin (Cat: 405232, dilution 1:1000). The following were purchased from eBioscience: anti-CD3 (Clone: 145-2C11, Cat: 11-0031-85, dilution 1:400), anti-NOS_2_ (Clone: CXNFT, Cat: 53-5920-82, dilution 1:100), anti-CD11b (Clone: M1/70, Cat: 47-0112-82 and 12-0112-82, dilution 1:200 or 1:1600), anti-CD11c (Clone: N418, Cat: 45-0114-82, dilution 1:200), anti-F4/80 (Clone: BM8, Cat: 17-4801-82 and 12-4801-82, dilution 1:200), anti-CD4 (Clone: GK1.5, Cat: 17-0041-82, dilution 1:1600), anti-CD45 (Clone: 30-F11, Cat: 47-0451-82, dilution 1:200), anti-CD90.2 (Clone: 53-2.1, Cat: 48-0902-82, dilution 1:400), Ly6C (Clone: HK1.4, Cat: 48-5932-82 and 25-5932-82, dilution 1:800), anti-mouse CD16/CD32 (Clone: 2.4G2, Cat: 14-0161-86, dilution 1:200), anti-CD25 (Clone: PC61.5, Cat: 102038, dilution 1:200), anti-ST2 (Clone: RMST2-2, Cat: 17-9335-82, dilution 1:100), biotin-labeled anti-CD19 (Clone: MB19-1, Cat: 13-0191-85, dilution 1:800), anti-CD11b (Clone: M1/70, Cat: 13-0112-85, dilution 1:800), anti-NK1.1 (Clone: PK136, Cat: 13-5941-82, dilution 1:800), anti-ter119 (Clone: TER-119, Cat: 13-5921-85, dilution 1:800), anti-B220 (Clone: RA3-6B2, Cat: 13-0452-85, dilution 1:6400). Streptavidin microbeads (Cat: 130-048-101) were purchased from Miltenyi Biotec. Purified neutralizing antibodies to CCL17/TARC (Cat: AF529) and CCL22/MDC (Cat: AF439), were obtained from R&D Systems. Purified blocking antibody against CD4 (Clone GK1.5) was from BioXcell. TGF-β1 neutralizing antibody (Cat: MAB240) was from R&D Systems.

### Construction of bone marrow chimeras

For *NOS*_2_^−/−^ and C57BL/6 chimera mice preparation, C57BL/6 or *NOS*_2_^−/−^ hosts were firstly irradiated with an X-ray animal irradiator (Rad Source RS2000) with 2 doses of 5.5 Gy (3–4 h apart). Then, 2 × 10^6^ bone marrow cells from *NOS*_2_^−/−^ or C57BL/6 mice were i.v. transferred into these lethally irradiated C57BL/6 or *NOS*_2_^−/−^ mice. 0.5 mg per ml neomycin (Sigma-Aldrich) was provided for the first 3 days after irradiation. Mice were employed in the study 8 weeks later.

### Depletion of lineage-specific cells in vivo

For depletion of lung CD11c^+^ cells in vivo, *CD11c-DTR* Tg mice or C57BL/6 mice received an i.t. injection of diphtheria toxin (100 ng per mouse) 1.5 days before the first OVA challenge. For depletion of lung cDC1s, *langerin-DTR* mice received an intraperitoneal injection of diphtheria toxin (1 mg per mouse) 1 day before the first OVA or papain challenge. In the AM depletion experiments, clodronate (dichloromethylene bisphosphonate, Cl2MBP) or PBS encapsulated liposomes (Encapsula Nanosciences) were injected intratracheally (60 μl, 1:2 dilution in PBS) to C57Bl/6 mice recipient 1 day before the first OVA challenge. In the NO inhibitor experiments, 1 day prior to the first challenge, mice received 400 μg 1400 W by i.p. injection and daily twice more from day −1 to day 1. Control mice received the same volume of saline.

### Kinetic model of allergic airway inflammation

Mice were sensitized by i.p. injection of 20 μg OVA (Grade VI) emulsified in 2.25 mg of Imject Alum in a total volume of 100 μl, on days −28 and −14, and then challenged (20 min) via the airways with OVA (Grade V; 1% in saline) on day 0, day 1, and day 2 with ultrasonic nebulization (PARIBOY SX, Germany), were culled 0.5 days (12 h), 1.5 days (36 h), 2.5 days (60 h), 4.5 days (108 h), 6.5 days (156 h), 8.5 days (204 h) after the first OVA challenge, and bronchoalveolar lavage fluid (Balf) and lungs were collected for analysis. Mice were culled 1.5 days (36 h) after the first OVA challenge in most of the OVA-induced allergic airway inflammation model, except in indicated experiments in Fig. 1e (middle and right), Fig. 5a, and Fig. 5c−h,  in which mice were culled 2.5 days (60 h) after the first OVA challenge. Sensitized mice challenged with saline were used as controls.

### Papain-induced allergic airway inflammation

Mice were sensitized with 30 μg papain each time at intervals days −28, −27, and −14, then i.n. challenged with 10 μg papain on day 0, and analyzed on day 1. See ref. ^58^ upon which this was based, with some modifications. Sensitized mice challenged with saline were used as controls.

### Balf

For Balf, the trachea was cannulated and the lungs were lavaged 2 times with 0.5 ml PBS. Eosinophils in the Balf were analyzed by FACS.

### Preparation of lung single cell suspensions

Mice were then killed, and lung parenchyma was collected and digested with 1 mg per ml collagenase IV for 1 h at 37 °C. Tissues were filtered through a 70 μm cell strainer, and resuspended in 30% Percoll for centrifugation at 1200*g* for 20 min at room temperature, followed by incubation with ACK buffer to lyse erythrocytes. All isolated cells were suspended in PBS supplemented with 2 mM EDTA and 1% FBS. In some indicated experiments, lung single cells were prepared without Percoll treatment for sorting CD45^+^ or CD45^−^ cells.

### Flow cytometry and sorting

Lung, Balf, and bone marrow (BM) cells were first blocked with 2.4G2 to eliminate of Fc receptor-mediated antibody binding. Cells were then incubated for 20 min on ice with antibodies. Cells were examined by flow cytometry using the Fortessa or Aria II Flow Cytometer (BD Bioscience) and analyzed with FlowJo software 10.0 (Tree Star). Cell sorting was performed on the Aria II Flow Cytometer (BD Bioscience).

The cells were sequentially gated using the following makers: cDC1s (CD64^−^F4/80^−^SiglecF^−^CD11c^+^IA/IE^+^CD103^+^CD11b^−^), CD24^+^ cDC2s (CD64^−^F4/80^−^SiglecF^−^CD11c^+^IA/IE^+^CD103^−^CD11b^+^CD24^+^), CD24^−^CD11b^+^ DC2s (CD64^−^F4/80^−^SiglecF^−^CD11c^+^IA/IE^+^CD103^−^CD11b^+^CD24^−^), MCs (CD64^+^F4/80^+^IA/IE ^int^CD11c^int^), IMs (CD64^+^F4/80^+^CD11c^low^), CD11b^+^ DCs (SiglecF^−^CD11c^+^IA/IE^+^CD103^−^CD11b^+^). In some indicated experiments, cDC1s were sequentially gated using the following makers (SiglecF^−^CD11c^+^IA/IE^+^CD103^+^CD11b^−^).

### Enzyme-linked immunosorbent assay

Cytokine and chemokine concentrations in cell-free Balf were measured with Multiplex reagents (Millipore). CCL17/TARC and CCL22/MDC in the Balf were analyzed using ELISAs (R&D Systems, Minneapolis, MN) specific for mouse CCL17/TARC (Catalog Number: MCC170) and mouse CCL22/MDC (Catalog Number: MCC220) according to the manufacturer’s protocols.

### Griess assay

Nitrite production was assayed by measurement of the nitrite ion concentration with the Griess assay (Beyotime Institute of Biotechnology, Shanghai, China) according to the manufacturer’s protocol. Lung CD45^+^ cells (1 × 10^6^ cells), cDC1s, CD24^−^CD11b^+^ DC2s, CD24^+^ cDC2s, MCs, AMs and IMs (5 × 10^4^ cells) were sorted from mice 1.5 days after the first OVA challenge and cultured for 48 h. NO production was measured in the supernatants of the cultured cells.

### Q-PCR

For quantitative PCR analysis, total RNA from sorted cells was extracted using RNeasy Mini Kit (Qiagen). cDNA was synthesized according to the manufacturers’ instructions through a QuantiTect Reverse Transcription Kit (Qiagen). Q-PCR was performed with the SYBR Green Master Mix (Qiagen) using the Rotor-Gene Q (Qiagen) or LightCycler480 (Roche) according to the manufacturer’s protocol. Target gene expression was calculated using the comparative method for relative quantification after normalization to *GAPDH* gene expression. The sequences for primers were as follows: *CCL5* (5′-*GCTGCTTTGCCTACCTCTCC*-3′ reverse, 5′-*TCGAGTGACAAACACGACTGC*-3′); *CCL8* (forward, 5′-*TCTACGCAGTGCTTCTTTGCC*-3′; reverse, 5′-*AAGGGGGATCTTCAGCTTTAGTA*-3′); *CCL11* (forward, 5′-*GAATCACCAACAACAGATGCAC*-3′; reverse, 5′-*ATCCTGGACCCACTTCTTCTT*-3′); *CCL17* (forward, 5′-*TACCATGAGGTCACTTCAGATGC*-3′; reverse, 5′-*GCACTCTCGGCCTACATTGG*-3′); *CCL22* (forward, 5′-*AGGTCCCTATGGTGCCAATGT*-3′; reverse, 5′-*CGGCAGGATTTTGAGGTCCA*-3′); *CCL24* (forward, 5′-*ATTCTGTGACCATCCCCTCAT*-3′; reverse, 5′-*TGTATGTGCCTCTGAACCCAC*-3′); *NOS*_2_ (forward, 5′-*TCAACATCTCCTGGTGGAAC*-3′; reverse, 5′-*AGCACACATGCAGAATGAGTA*-3′); *TGF-β1* (forward, 5′-*ACCATGCCAACTTCTGTCTG*-3′; reverse, 5′-*CGGGTTGTGTTGGTTGTAGA*-3′); and GAPDH (forward, 5′-TGTGTCCGTCGTGGATCTGA-3′; reverse, 5′-*TTGCTGTTGAAGTCGCAGGAG*-3′).

### Air-pouch assay

The subcutaneous air-pouch model of DC-induced eosinophilic inflammation was used^36,37^. Subcutaneous air pouches were generated by injection of 3 ml of air into the subcutaneous tissue on the back of anesthetized female mice. Air pouches were reinflated 3 days later. The next day, CD45^−^ cells or CD45^+^ cells, lung CD4^+^ T cells, CD45^+^lin^−^CD90.2^+^CD127^+^CD25^+^ST2^+^ ILC2s, or DC subsets (1–5 × 10^4^ cells), were injected into the pouches; 12 h later, pouches were washed with 3 ml cold PBS. CD11b^+^IA/IE^−^SiglecF^hi^SSC^hi^ eosinophil counts were obtained with FACS analysis. For some experiments, lung CD45^−^ cells or CD45^+^ cells (left, 1 × 10^6^) were sorted from mice 1.5 days after the first OVA challenge; lung CD4^+^ T cells (middle, 1 × 10^5^) or ILC2s (right, 1 × 10^4^) were sorted from mice 1.5 days and 2.5 days after the first OVA challenge. In certain recruitment assays, lung DC subsets were mixed with 2.5 μg per ml anti-CCL17/TARC (Fig. 3e), 1.5 μg per ml anti-CCL22/MDC, or 10 μg per ml anti-TGF-β1 blocking antibody in the air pouches. In certain experiments, CCL17 (150 pg, 500 μl) or CCL22 (150 pg, 500 μl) in a temperature-sensitive surface gel was injected into the air pouches, and 5 h later, eosinophils recruited into the air pouches were assessed.

### Transwell assay

The direct recruitment of lung eosinophils was assayed using Transwell^TM^ inserts (pore size 3 μm) and 24-well culture plates (Corning Costar, Cambridge, MA). Briefly, bone marrow Gr1^−^F4/80^+^SSC^high^ eosinophils were sorted from challenged mice with a FACSAria II flow cytometer and then labeled with 0.1 μmol CFSE. 2 × 10^5^ CFSE-labeled BM eosinophils in 0.2 ml RPMI-1640 medium containing 10% FBS were transferred to the upper compartment of the transwell insert. Lung cDC1s, CD24^+^ DC2s, or MCs (1.5 × 10^4^) were sorted (FACSAria II) from challenged C57BL/6 mice 1.5 days after the first OVA challenge and were added in the lower compartment of the transwell insert. RPMI-1640 containing 10% FBS was used as a control. After 12 h incubation at 37 °C in an atmosphere with 5% CO_2_, the number of CFSE^+^ eosinophils that had migrated from the upper to the lower compartment was counted using FACS. In certain experiments, CCL17 (100 pg per ml) or CCL22 (100 pg per ml) were added in the lower compartment of the transwell insert. 2 × 10^5^ CFSE-labeled bone marrow eosinophils were transferred to the upper compartment of the transwell insert. After 2 h incubation at 37 °C and 5% CO_2_, the number of CFSE^+^ eosinophils that had migrated from the upper to the lower compartment was counted using FACS.

### Adoptive transfer

To test the capacities of lung cDC1s, CD24^+^ DC2s, or MCs from challenged mice to induce eosinophil infiltration, we performed an adoptive transfer experiment. Lung cDC1s, CD24^+^ DC2s, or MCs (5 × 10^4^ cells, 50 μl, *n* = 3–4 mice per group) were sorted from mice 1.5 days after the first OVA challenge using BD FACSAria II Flow Cytometer and were administered i.t. into sensitized C57B/6 mice on day 0. Mice were culled 48 h after adoptive transfer for analysis of lung eosinophil infiltration.

### Statistics

For all relevant animal experiments, age-matched female mice were randomly chosen to be in different treatment groups. Each group was typically composed of three to nine (except for the *NOS*_2_^−/−^ chimeric mice experiment, which was composed of 6–14) mice 8–12 weeks of age, and two to three independent experiments were performed for every assay. Statistical analysis was performed in GraphPad Prism software 6.0. Data were analyzed by application of two-tailed unpaired Student’s *t* test where necessary. A *P* value of less than 0.05 was considered significant.

## Electronic supplementary material

Supplementary Information

## Data Availability

The data that support this study are available within the article and its Supplementary Information files or available from the authors upon reasonable request.
