# Supplementary material for: Eosinophil recruitment is dynamically regulated by interplay among lung dendritic cell subsets after allergen challenge
Source: Nat Commun. 2018 Sep 24;9:3879. doi: 10.1038/s41467-018-06316-9 (PMC6155158; doi:10.1038/s41467-018-06316-9)
Supplement: Supplementary file 1 — Supplementary Information [file 41467_2018_6316_MOESM1_ESM.pdf]

Eosinophil recruitment is dynamically regulated by interplay among lung dendritic cell subsets after allergen challenge

Yi et al.

Supplementary Information

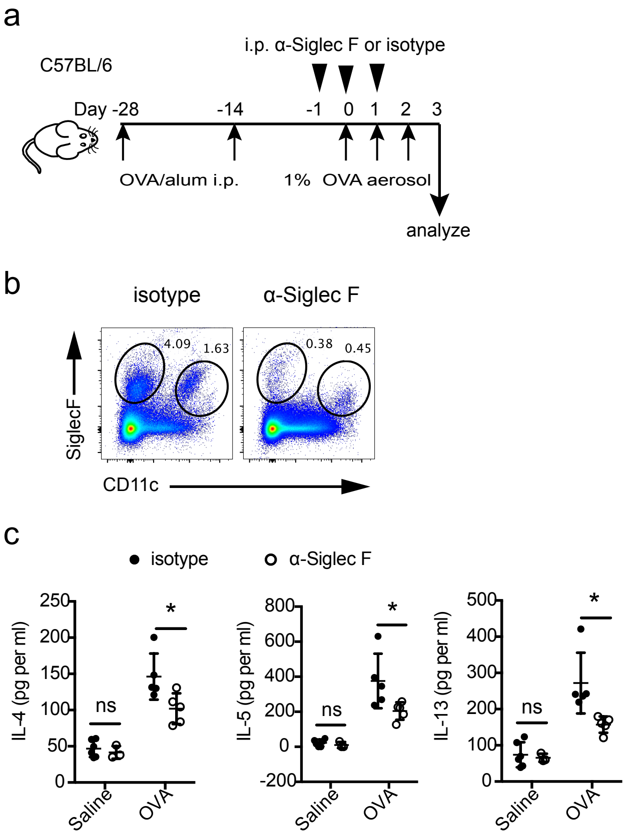

Supplementary Figure 1. Allergic response of B6 mice after α-Siglec-F treatment. (a) One day prior to the first challenge and daily for 2 additional doses on day 0 and day 1, mice received 20 μg monoclonal antibody to Siglec-F (E50-2440, BD) or the same volume of rat IgG2a isotype control by i.p. injection. (b) Different deletion efficiencies of eosinophils and AMs in the lungs of C57Bl/6 mice after α-Siglec F treatment. (c) ELISA analysis of IL-4, IL-5, and IL-13 in Balf supernatant from mice after α-Siglec F (solid circle)or isotype control (empty circle) treatment. n = 5–6 mice per group except α-Siglec F-treated saline control mice, with n = 3 mice. *P < 0.05, unpaired Student's *t* test. Means ± SD are shown. Data represent two independent experiments.

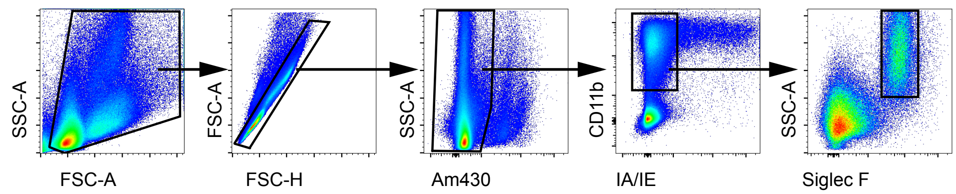

Supplementary Figure 2. Identification of eosinophils in air-pouch assay. Cells sorted by FACS from lungs were injected into the air-pouches on the backs of naïve mice. Twelve hours later, cells were obtained from the air pouches, $CD11b^{+}IA/IE^{-}SiglecF^{hi}SSC^{hi}$ cells were identified as eosinophils and were counted by FACS.

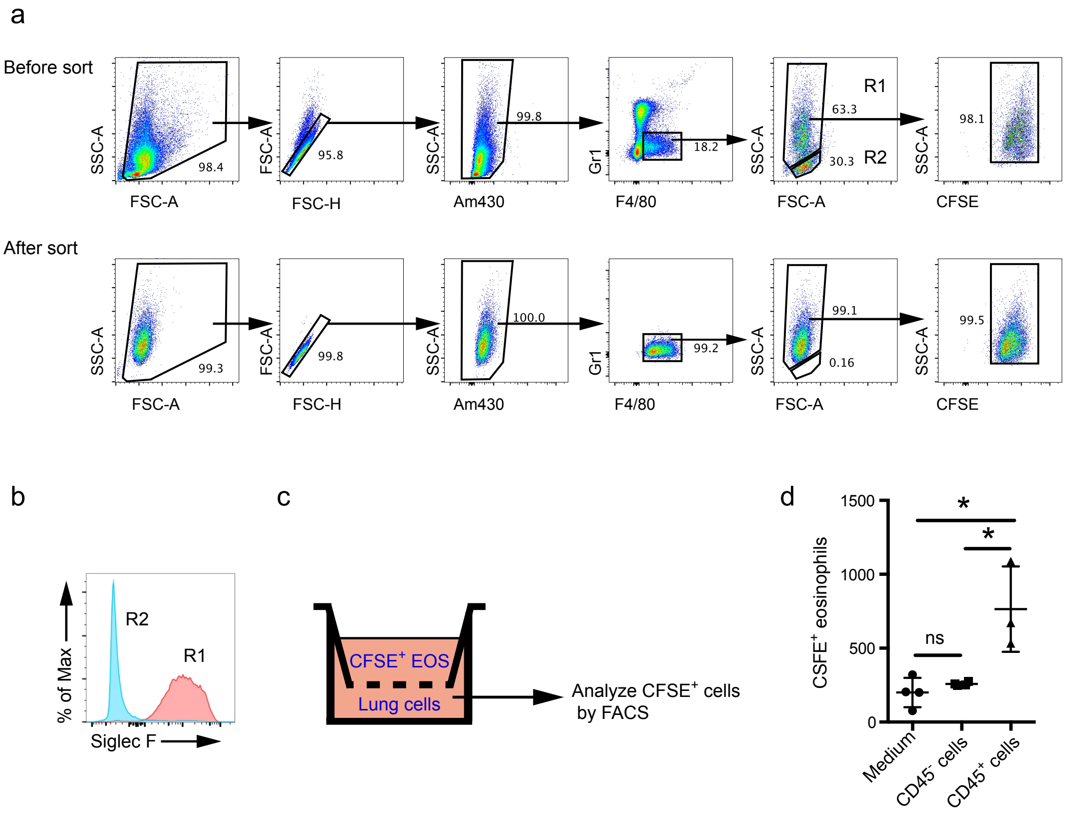

Supplementary Figure 3. The direct chemotactic effect of lung CD45^{+} cells. (a) Gating strategy used to identify eosinophils in mouse bone marrow before and after sorting. The strategy to identify bone marrow eosinophils was described previously with some modifications [1]. To avoid anti-siglec F crosslink mediated-apoptosis of eosinophils [2], F4/80 antibody was used to replace anti-siglec F antibody to identify bone marrow eosinophils, as Gr1^{-}F4/80^{+}SSC^{high} bone marrow cells were exclusively Siglec F-positive (Supplementary Figure 3b). Therefore, bone marrow Gr1^{-}F4/80^{+}SSC^{high} cells were sorted from challenged mice with FACS as eosinophils and then labeled with 0.1 μmol CFSE. Lung CD45^{-} cells or CD45^{+} cells (1 ×10^{6}) sorted from C57BL/6 mice lungs 1.5 d after the first OVA challenge were seeded in the lower chamber of the transwell system (c), while CFSE-labeled bone marrow eosinophils were on the upper inserts. (d) Twelve hours later, CFSE^{+} eosinophils in the lower chamber were calculated by FACS. n = 3–4 per group. *$P < 0.05$, unpaired Student's *t* test. Means ± SD are shown. Data represent two independent experiments.

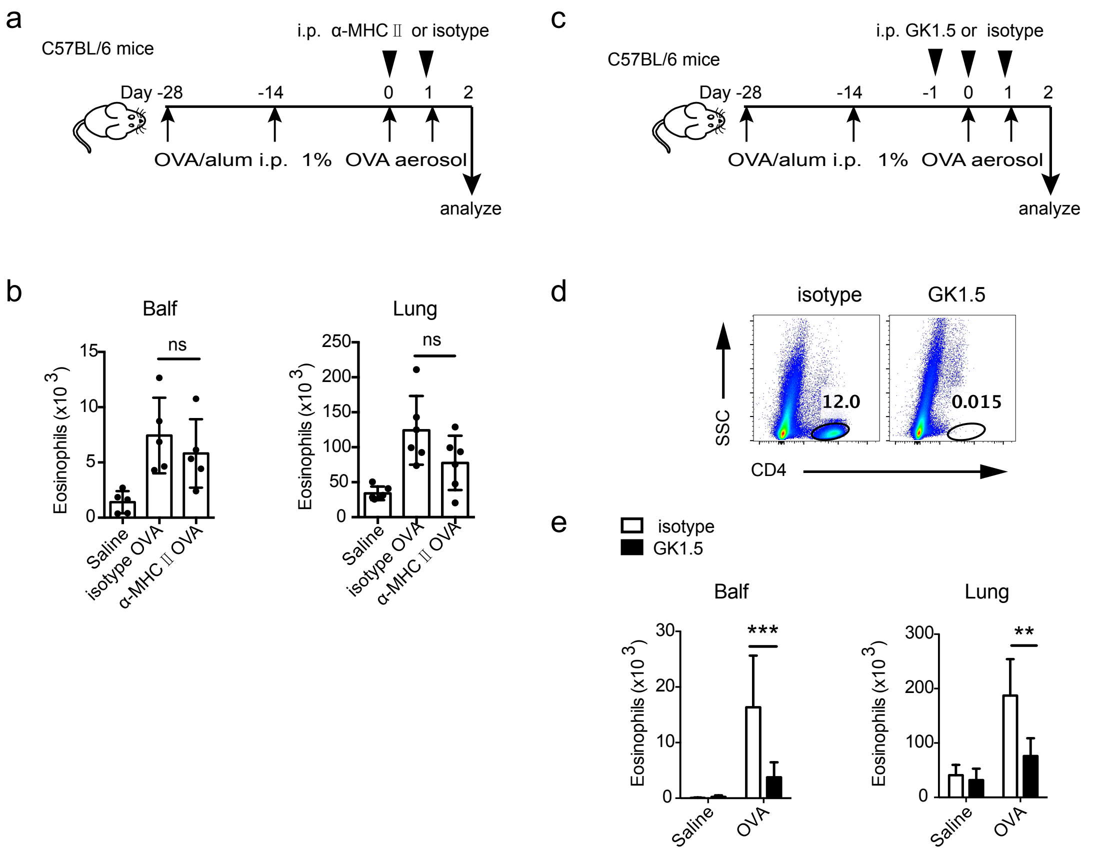

Supplementary Figure 4. CD4^{+} T cells are required for eosinophil infiltration. (a) The mouse model of α-MHC II treatment. One hour before OVA challenge, mice received 500 μg α-MHC II (clone: Y-3P; Bio-X Cell) or mouse IgG2a isotype control by i.p. injection daily for 2 times from d 0 to d 1. (b) Total number of eosinophils in the Balf and lungs were assessed in mice 1.5 d after the first OVA challenge as shown in (a). n = 5–6 per group. (c) The mouse model of anti-CD4 treatment. Twenty-four hours before the first OVA challenge, mice received 300 mg anti-CD4 mAb (clone GK1.5; Bio-X Cell) or rat IgG2b isotype control by intravenous injection daily three times from day −1 to day 1. (d) Deletion efficiency of CD4^{+} T cells in the lungs of wild-type mice 24 hr after 300 mg anti-CD4 mAb. (e) Total number of eosinophils in the Balf or lungs was obtained from anti-CD4 mAb (solid rectangle) or isotype control (empty rectangle) treated mice 1.5 d after the first OVA challenge. n = 5–6 per group. *P < 0.05, **P < 0.01, ***P < 0.001, unpaired Student's *t* test. Means ± SD are shown. Data represent two (Supplementary Figure 4a, b) three (Supplementary Figure 4c-e) independent experiments.

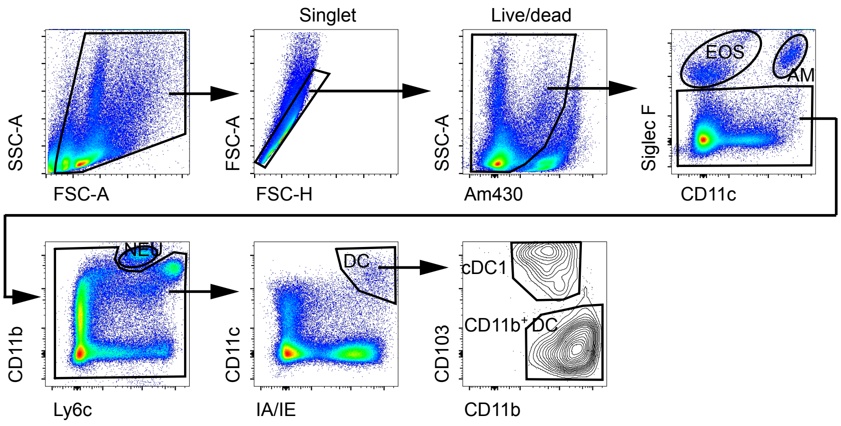

Supplementary Figure 5. Gating strategy for identification of lung myeloid cells. Cells were isolated from enzymatically digested normal mouse lungs. Sequential gating strategy to identify lung myeloid cell subsets was defined in published reports. After the exclusion of doublets and debris, dead cells were excluded at this step, using live/dead staining. In normal mouse lungs, AMs and eosinophils were readily identified, based on the expression of Siglec F, CD11c. Neutrophils were identified based on their expression of Ly6c and CD11b ($Ly6c^{int}$ $CD11b^{+}$). Remaining cells were separated using CD11c and MHCII. Two populations of DCs were identified on the basis of CD103 and CD11b expression: cDC1 and $CD11b^{+}DC$. In some indicated experiments (Fig. 2h-i, Fig. 3e, Fig. 4e, Fig. 4j, Fig.5 h and supplementary Fig. 10), cDC1s and $CD11b^{+}DC$ were sequentially gated using the following makers: cDC1s ($SiglecF^{-}CD11c^{+}IA/IE^{+}CD103^{+}CD11b^{-}$), $CD11b^{+}DC$ ($SiglecF^{-}CD11c^{+}IA/IE^{+}CD103^{-}CD11b^{+}$),

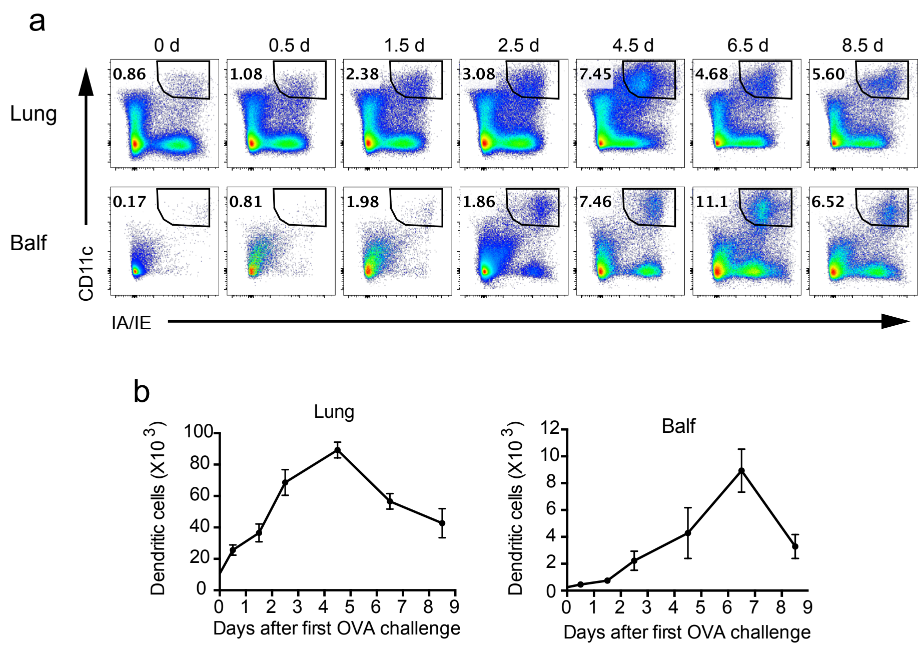

Supplementary Figure 6. Temporal changes of DCs in allergic mice. FACS analysis (a) and total numbers (b) of kinetics of DC recruitment in different lung compartments in the murine model of allergic inflammation (the same as Fig.1a). Upper row in (a), lung tissue homogenates; lower row in (a), Balf (matching animals). n = 4–8 mice per group. Data represent two independent experiments.

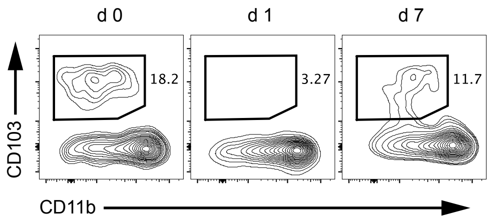

Supplementary Figure 7. FACS analysis of lung cDC1 in langerin-DTR mice. Langerin-DTR mice received an intraperitoneal injection of diphtheria toxin (1 mg per mouse) on day 0. The depletion of lung cDC1s was analyzed in langerin-DTR mice on days 1 and 7.

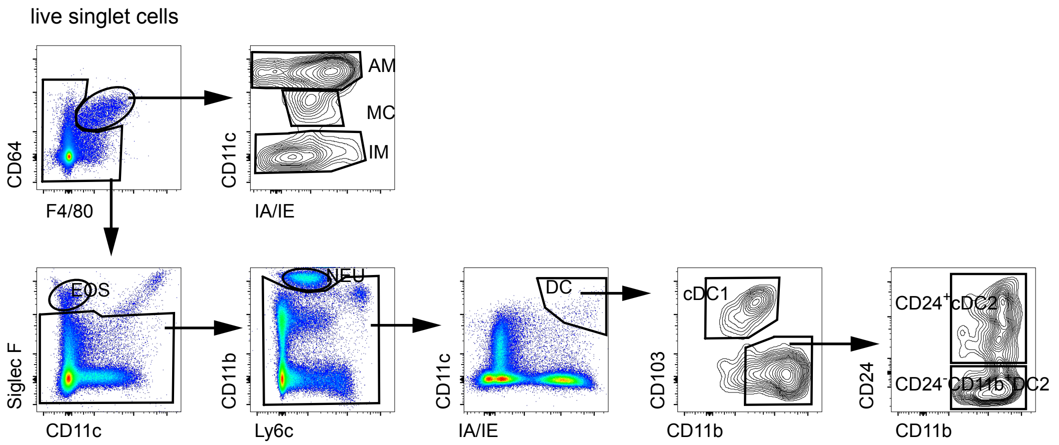

Supplementary Figure 8. Gating strategy for identification of lung DC subsets. Cells were isolated from enzymatically digested normal mouse lungs. AMs, MCs and IMs were identified based on the expression of CD11c and MHCII in live singlet $CD64^+$ $F4/80^+$ cells. Remaining cells were separated using the same strategy as shown in Supplementary Figure 4. Three populations of DCs were identified on the basis of CD103, CD11b, and CD24 expression: cDC1, $CD24^+$cDC2, and $CD24^-CD11b^+$ DC2. Most experiments carried out with this gating strategy, which carefully gated on cDC2 excluding monocyte derived cells (Fig. 2j,k,l; Fig. 3a; Fig. 4f,g,i, Fig.5c,d,e,f,g; supplementary Fig. 12,13.). The cells were sequentially gated using the following makers: cDC1s ($CD64^-F4/80^-SiglecF^-CD11c^+IA/IE^+CD103^+CD11b^-$), $CD24^+$ cDC2s ($CD64^-F4/80^-SiglecF^-CD11c^+IA/IE^+CD103^-CD11b^+CD24^+$), $CD24^-CD11b^+$ DC2s ($CD64^-F4/80^-SiglecF^-CD11c^+IA/IE^+CD103^-CD11b^+CD24^-$), MC ($CD64^+F4/80^+IA/IE^{int}CD11c^{int}$), IM ($CD64^+F4/80^+CD11c^{low}$).

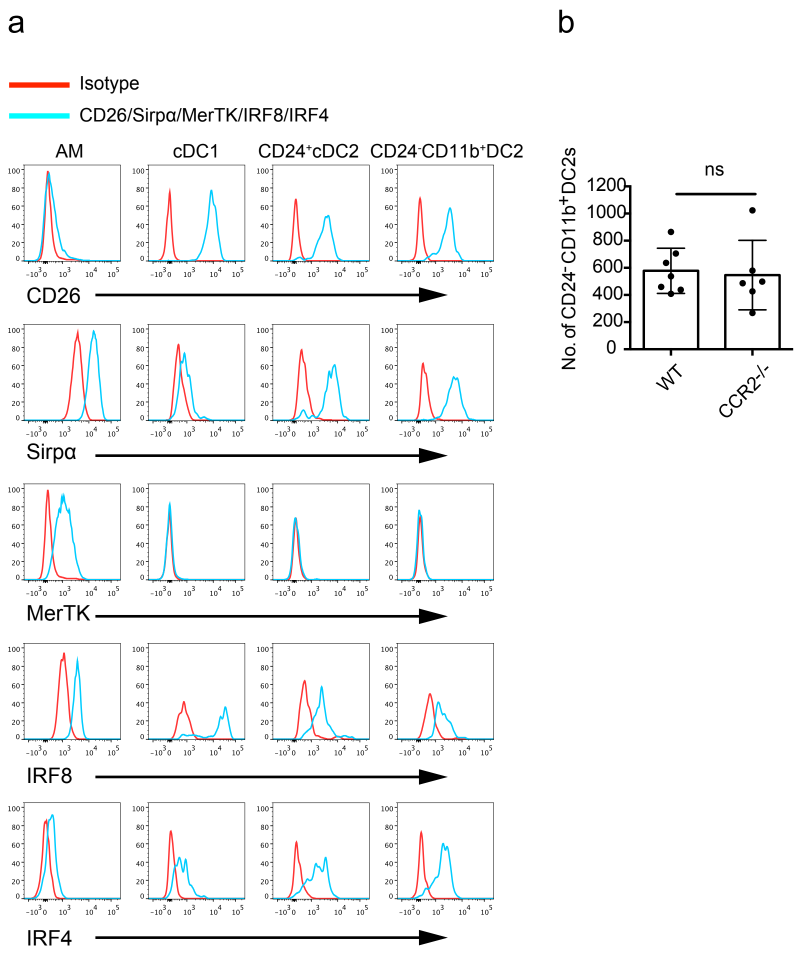

Supplementary Figure 9. Characterization of lung CD24^{-}CD11b^{+} DC2s. (a) The cells were sequentially gated using the following makers: AMs (CD64^{+}F4/80^{+}CD11c^{hi}IA/IE$^{low\ to\ int}$), cDC1s (CD64^{-}F4/80^{-}SiglecF^{-}CD11c^{+}IA/IE^{+}CD103^{+}CD11b^{-}), CD24^{+} cDC2s (CD64^{-}F4/80^{-}SiglecF^{-}CD11c^{+}IA/IE^{+}CD103^{-}CD11b^{+}CD24^{+}), CD24^{-}CD11b^{+} DC2s (CD64^{-}F4/80^{-}SiglecF^{-}CD11c^{+}IA/IE^{+}CD103^{-}CD11b^{+}CD24^{-}). Cells were subsequently analyzed for the expression of CD26, Sirpa, Mertk, IRF8 and IRF4 expression. Histograms show different markers (blue line) and isotypes (red line). Cells were examined by Aria III Flow Cytometer (BD bioscience), (b) The number of lung CD24^{-}CD11b^{+} DC2s population was not affected in CCR2-deficient mice. n = 6–7 per group. *P < 0.05, unpaired Student's *t* test. Means ± SD are shown. Data represent two independent experiments.

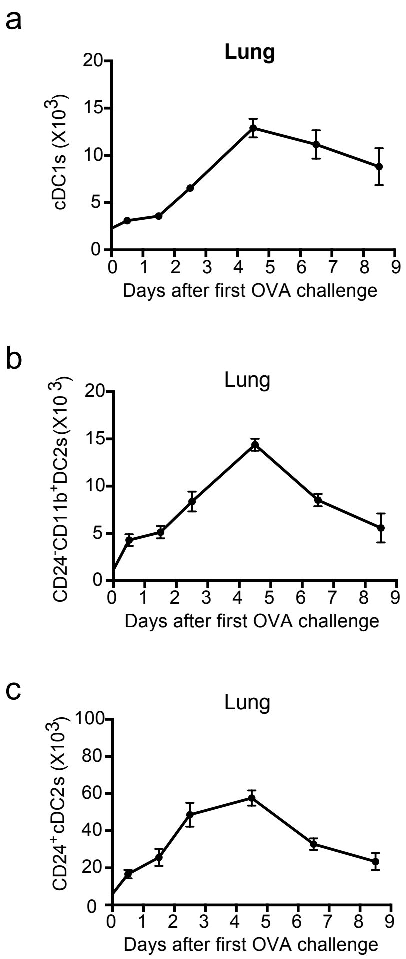

Supplementary Figure 10. Temporal changes of lung DC subsets in allergic mice. Total numbers of kinetics of cDC1s (SiglecF^{-}CD11c^{+}IA/IE^{+}CD103^{+}CD11b^{-}), CD24^{+} cDC2s (SiglecF^{-}CD11c^{+}IA/IE^{+}CD103^{-}CD11b^{+}CD24^{+}) and CD24^{-}CD11b^{+} DC2s (SiglecF^{-}CD11c^{+}IA/IE^{+}CD103^{-}CD11b^{+}CD24^{-}) recruitment in lung compartments in the murine model of allergic inflammation (the same as in Fig. 1a). n = 4–6 mice per group. Data represent two independent experiments.

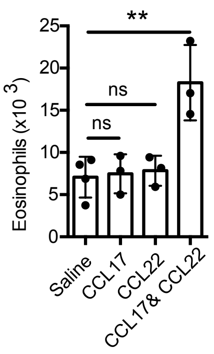

Supplementary Figure 11. Air-pouch assay of CCL17 or CCL22 with low concentrations. Counts of eosinophils recruited into the air pouches of wild-type mice 5 hr after injection of CCL17 or/and CCL22 (50 pg each) with 200 μl temperature-sensitive surface gel. n = 5–6 mice per group. *P < 0.05, **P < 0.01, unpaired Student's *t* test. Means ± SD are shown. Data represent two independent experiments.

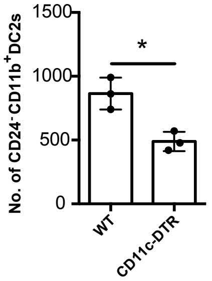

Supplementary Figure 12. Depletion of lung $CD24^{-}CD11b^{+}$ DC2s in CD11c-DTR mice. *CD11c-DTR* Tg mice or C57BL/6 mice (WT) received an i.t. injection of DT (100 ng per mouse). The depletion of lung $CD24^{-}CD11b^{+}$ DC2s was analyzed in the lungs 1.5 days later. n = 3 mice per group. *P < 0.05, unpaired Student's *t* test. Means ± SD are shown. Data represent two independent experiments.

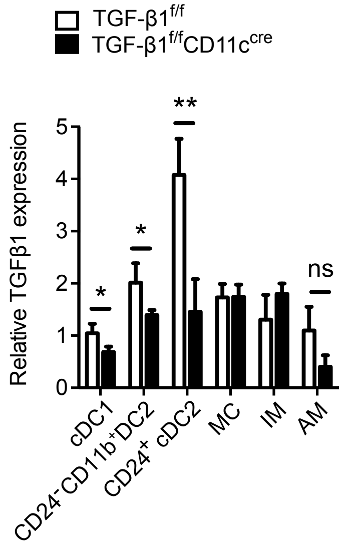

Supplementary Figure 13. mRNA expression of TGF-β1 in CD24^{+} cDC2s. mRNA expression of TGF-β 1 by pulmonary cDC1s, CD24+ cDC2s, CD24−CD11b+ DC2s, MC, IM and AM populations separated from TGF-β 1$^{fl/fl}$CD11c^{Cre} (solid rectangle) and TGF-β1$^{fl/fl}$ (empty rectangle) mice 2.5 d after the first OVA challenge. n = 3–4 per group. *P < 0.05, **P < 0.01, unpaired Student's *t* test. Means ± SD are shown. Data represent two independent experiments.

Supplementary References

1. Chu, V.T.*, et al.* Eosinophils are required for the maintenance of plasma cells in the bone marrow. *Nature immunology* **12**, 151-159 (2011).
2. Mao, H.*, et al.* Mechanisms of Siglec-F-Induced Eosinophil Apoptosis: A Role for Caspases but Not for SHP-1, Src Kinases, NADPH Oxidase or Reactive Oxygen. *Plos One* **8**(2013).
